# Extensive population genetic structure in the giraffe

**DOI:** 10.1186/1741-7007-5-57

**Published:** 2007-12-21

**Authors:** David M Brown, Rick A Brenneman, Klaus-Peter Koepfli, John P Pollinger, Borja Milá, Nicholas J Georgiadis, Edward E Louis, Gregory F Grether, David K Jacobs, Robert K Wayne

**Affiliations:** 1 Department of Ecology and Evolutionary Biology, University of California, Los Angeles, CA, 90095, USA; 2 Center for Conservation and Research, Omaha's Henry Doorly Zoo, 3701 South 10th Street, Omaha, NE 68107, USA; 3 Mpala Research Centre, PO Box 555, Nanyuki, Kenya

## Abstract

**Background:**

A central question in the evolutionary diversification of large, widespread, mobile mammals is how substantial differentiation can arise, particularly in the absence of topographic or habitat barriers to dispersal. All extant giraffes (*Giraffa camelopardalis*) are currently considered to represent a single species classified into multiple subspecies. However, geographic variation in traits such as pelage pattern is clearly evident across the range in sub-Saharan Africa and abrupt transition zones between different pelage types are typically not associated with extrinsic barriers to gene flow, suggesting reproductive isolation.

**Results:**

By analyzing mitochondrial DNA sequences and nuclear microsatellite loci, we show that there are at least six genealogically distinct lineages of giraffe in Africa, with little evidence of interbreeding between them. Some of these lineages appear to be maintained in the absence of contemporary barriers to gene flow, possibly by differences in reproductive timing or pelage-based assortative mating, suggesting that populations usually recognized as subspecies have a long history of reproductive isolation. Further, five of the six putative lineages also contain genetically discrete populations, yielding at least 11 genetically distinct populations.

**Conclusion:**

Such extreme genetic subdivision within a large vertebrate with high dispersal capabilities is unprecedented and exceeds that of any other large African mammal. Our results have significant implications for giraffe conservation, and imply separate *in situ *and *ex situ *management, not only of pelage morphs, but also of local populations.

## Background

**Figure 1:**
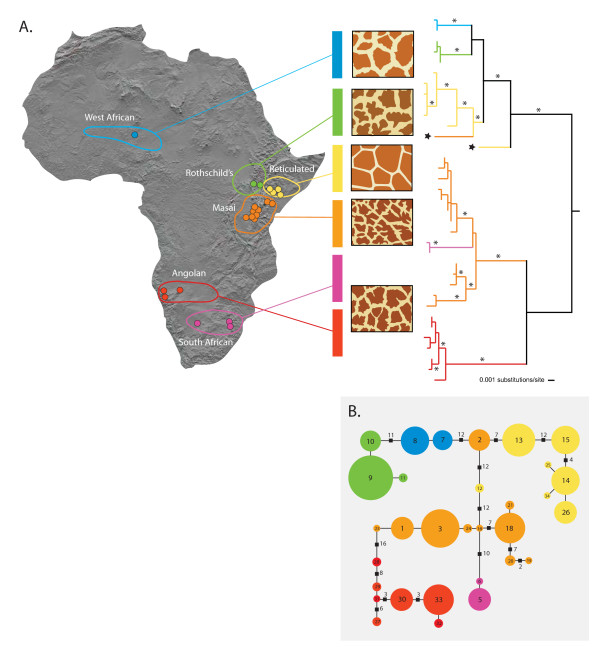
**Genetic subdivision in the giraffe based on mitochondrial DNA sequences**. (A) Approximate geographic ranges, pelage patterns, and phylogenetic relationships between giraffe subspecies based on mtDNA sequences. Colored dots on the map represent sampling localities (see Additional files 1 and 10 for detailed locality information). The phylogenetic tree is a maximum-likelihood phylogram based on 1707 nucleotides of mtDNA sequence (1143 nt of cytochrome *b*, 429 nt control region and 135 nt of tRNA) from 266 giraffes. Asterisks along branches correspond to node-support values of > 90% bootstrap support. Stars at branch tips identify paraphyletic haplotypes found in Masai and reticulated giraffes. (B) Minimum-spanning network of control region haplotypes using the molecular-variance parsimony algorithm (see Additional file 8), where circles represent haplotypes, numbers within them correspond to haplotype designations, and circle sizes are proportional to the haplotype's frequency in the population. Branches represent a single nucleotide change and black squares represent multiple changes (indicated by adjacent numbers). Colors are coded as in Figure 1A.

In highly mobile species that are distributed across continuous habitat, persistent gene flow can stifle genetic differentiation and speciation [1]. Adult giraffes (*Giraffa camelopardalis*) weigh in excess of 1000 kg [2], frequently range over several hundred square km and are capable of long distance movements of 50–300 km [3]. Further, giraffes live in loosely constructed social groups with large home range sizes, ranging from 5 km^2 ^to 992 km^2 ^[3,4] throughout scrub and savannah habitat from the Sahel to South Africa (Figure 1A). These life history attributes would predict that low levels of differentiation should be found among populations because the extent of gene flow is related to the dispersal potential of individuals [5]. Consistent with this prediction, large-bodied mammals such as the African elephant (*Loxodonta africana*) and African buffalo (*Syncerus caffer*) generally have low levels of differentiation between adjacent populations [6,7]. However, giraffes exhibit a marked pattern of geographic variation in pelage coloration (Figure 1A) as well as in ossicone number [8,9] and mitochondrial variation [10] suggesting significant population differentiation despite the potential for high rates of genetic exchange.

Within the last century, numerous taxonomic schemes have been developed to reflect the regional differences in pelage pattern and morphology. These schemes have ranged from the recognition of two species, *G. reticulata *and *G. camelopardalis *and 10 subspecies for the latter [8], to the recognition of a single species (*G. camelopardalis*), with nine [11], eight [12], six [13] or five [14] subspecies (Table 1). The controversy regarding giraffe subspecies in part reflects high variability in pelage patterns within some populations [15] and suspected hybridization among putative subspecies [2,16,17]. Nonetheless, the boundaries between pelage types and the subspecies they represent can be abrupt and do not necessarily correspond to apparent habitat or topographic obstacles to dispersal. For example, in Kenya, Masai (*G.c. tippleskirchi*), reticulated (*G.c. reticulata*), and Rothschild's giraffes (*G.c. rothschildi*) have geographic boundaries in continuous acacia scrub woodland habitat [2]. The marked geographic differences in characters such as pelage pattern (Figure 1A) suggest reproductive isolation despite the potential for genetic exchange, yet to date, no comprehensive genetic studies using nuclear and mitochondrial DNA markers have been conducted to evaluate this hypothesis.

**Table 1:** Taxonomic classifications proposed for the giraffe

**Author**	**Species**	**Subspecies**
Lydekker [8]	*Giraffa reticulata*	
	*G. camelopardalis*	*G. c. angolensis*
		*G. c. antiquorum*
		*G. c. capensis*
		*G. c. congolensis*
		*G. c. cottoni*
		*G. c. peralta*
		*G. c. rothschildi*
		*G. c. tippelskirchi*
		*G. c. typica*
		*G. c. wardi*
		
Dagg and Foster [11]	*G. camelopardalis*	*G. c. angolensis*
		*G. c. antiquorum*
		*G. c. camelopardalis*
		*G. c. giraffa*
		*G. c. peralta*
		*G. c. reticulata*
		*G. c. rothschildi*
		*G. c. thorncrofti*
		*G. c. tippelskirchi*
		
Kingdon [12]	*G. camelopardalis*	*G. c. angolensis*
		*G. c. camelopardalis*
		*G. c. congoensis*
		*G. c. giraffa*
		*G. c. peralta*
		*G. c. reticulata*
		*G. c. thorncrofti*
		*G. c. tippelskirchi*
		
East [13]	*G. camelopardalis*	"Western"^1^
		"Nubian/Rothschild's"^2^
		*G. c. reticulata*
		*G. c. thorncrofti*
		*G. c. tippelskirchi*
		"Southern"^3^
		
Grubb [14]	*G. camelopardalis*	*G. c. giraffa*
		*G. c. reticulata*
		*G. c. rothschildi*
		*G. c. thorncrofti*
		*G. c. tippelskirchi*

^1 ^includes *G. c. antiquorum *+ *G. c. congoensis *+ *G. c. peralta*.

^2 ^includes *G. c. camelopardalis *+ *G. c. rothschildi*.

^3 ^includes *G. c. angolensis *+ *G. c. capensis *+ *G. c. giraffa *+ *G. c. infumata *+ *G. c. wardi*.

In this study, we present a phylogeographic and population genetic analysis of the giraffe across most of the species' remaining geographic range. We sampled free-ranging giraffes representing six of the nine subspecies defined by Dagg and Foster [11] who used specific morphologic criteria and recognized five distinct pelage patterns (Table 1, Figure 1A). We assessed genetic variation in mitochondrial DNA (mtDNA) sequences and 14 nuclear microsatellite loci. We found concordant patterns of genetic subdivision in morphology and genetics coincident with subspecies boundaries and a fine scale pattern of genetic subdivision within putative subspecies. Such striking genetic partitioning within a highly mobile species is surprising, and implies environmental and behavioral mechanisms limit gene flow between populations. Our results have important conservation implications, as some of these genetically distinct populations clearly represent evolutionarily significant units (ESUs) that are highly threatened and lack appropriate recognition in current management plans.

## Results

### Analysis of the mitochondrial DNA sequences

We amplified and sequenced a 654-nucleotide (nt) fragment of mtDNA that spanned a region near the 3' end of the cytochrome *b *(*CYTb*) gene to the end of hypervariable region 1 of the control region in 266 giraffes from 19 localities (see Additional file 1) and one okapi (*Okapia johnstoni*). We detected 35 haplotypes that differed by 1–37 substitutions (uncorrected pairwise distance = 0.15–5.66%). To ensure phylogenetic resolution and robust support for relationships among haplotypes, the remaining portion of the *CYTb *gene was amplified and sequenced from 35 giraffes, representing each of the 35 unique haplotypes, and the okapi (1709 nt total). Phylogenetic analysis of these 35 sequences revealed the existence of seven primary clades that are well supported with bootstrap values generally over 90%. Moreover, these clades are largely consistent with pelage patterns and putative subspecific designations (Figure 1A). First, we found two West African haplotypes defined a cluster that is sister to a clade of three haplotypes belonging to the Rothschild's giraffes. This clade of West African and Rothschild's giraffe haplotypes is also supported by a synapomorphic single nucleotide insertion (A at position 350 of the 654-nt fragment) in the control region. These two clades are reciprocally monophyletic to a clade defined by the majority of haplotypes from the reticulated giraffe. A fourth and fifth clade are defined by Masai giraffe haplotypes east and west of the Rift Valley in Kenya. The Masai clade east of the Rift Valley is sister to a South African giraffe grouping defined by two haplotypes. Finally, a seventh clade is defined solely by Angolan giraffe haplotypes, which all share a synapomorphic T insertion at position 355 (of the 654-nt fragment) of the control region. The seven clades are grouped into two or three larger clades, depending on the method of rooting (Figure 1A; see Additional files 2, 3, 4, 5, 6, 7), although relationships among these larger clades was not well resolved. Notably, midpoint rooting, which places the root at the midpoint between the most divergent lineages (assuming a uniform rate of molecular evolution), divided giraffes into a northern group containing Western, Rothschild's and reticulated giraffes and a southern group containing Masai, Angolan and South African giraffes (Figure 1A).

Two haplotypes were paraphyletic with respect to their subspecies (stars at terminals in Figure 1A). One highly divergent haplotype was found in a single reticulated giraffe and formed a sister lineage to the clade containing West African, Rothschild's, and all other reticulated giraffe haplotypes. A second haplotype was found in nine Masai giraffes and clustered with the reticulated giraffe haplotypes. These isolated cases of paraphyly likely represent ancient introgression events [18] or incomplete lineage sorting of variants rather than recent gene flow given the congruence between subspecies and nuclear DNA data patterns (see below).

Divergence times between the seven clades obtained from coalescence analysis [19] ranged from 0.13–0.37 million years (MY) between Masai and South African clades, to 0.54–1.62 MY between the southern clade (Masai, Angolan and South African giraffes) and the northern clade (West African, Rothschild's and reticulated giraffes) (Table 2). Values for the northern giraffe grouping were intermediate, with West African and Rothschild's giraffes diverging about 0.16–0.46 MY ago, and the two splitting from reticulated giraffes about 0.18–0.54 MY ago. These dates argue for a mid to late Pleistocene radiation of giraffes.

**Table 2:** Divergence times between giraffe clades

				**Time (MY)**		
**Clades**	**Θ (SD)**	***M*(SD)**	***T*(SD)**	**0.05 s/s/MY**	**0.10 s/s/MY**	**0.15 s/s/MY**
[per] [rot]	0.55 (0.09)	0.01 (0.01)	18.10 (2.21)	0.464	0.232	0.155
[tip] [gir]	3.93 (0.24)	0.18 (0.03)	2.04 (0.11)	0.374	0.187	0.125
[per+rot] [ret]	2.72 (0.11)	0.04 (0.01)	4.26 (0.42)	0.540	0.270	0.180
[tip+gir] [ang]	5.57 (0.28)	0.04 (0.01)	3.32 (0.21)	0.862	0.431	0.287
[tip+gir+ang] [per+rot+ret]	5.31 (0.09)	0.05 (0.00)	6.72 (0.23)	1.617	0.808	0.539

Divergence times between giraffe clades in Figure 1 based on mtDNA control region sequence data. Maximum likelihood values of parameters θ, *M *(2N*m*), and *T *(*t*/2N) estimated with a non-equilibrium coalescence model using the program MDIV [19]. Time divergence values (in million years, MY) are given for three mutation rate estimates for the giraffe mtDNA control region (0.05, 0.10 and 0.15 substitutions per site per MY), and a generation time of 4 years. per = *G.c. peralta*, rot = *G.c. rothschildi*, ret = *G.c. reticulata*, tip = *G.c. tippelskirschi*, ang = *G.c. angolensis*, gir = *G.c. giraffa*.

Hierarchical analysis of molecular variance [20] based only on the mtDNA control region data (429 nt) corroborates the phylogenetic results (see Additional file 8). Groupings according to the six subspecies resulted in substantially higher values of genetic variance partitioned among groups (φ_ct _= 75.37, p < 0.001) than alternative groupings, corresponding to the deeper clades (see Additional file 8). Haplotype (*h*) and nucleotide (π) diversity of giraffe control region sequences were generally low except for Masai and reticulated giraffes, which had values an order of magnitude greater than other groups (Figure 1B; see Additional file 9). The higher mtDNA diversity in both Masai and reticulated giraffes and the central placement of their haplotypes in the minimum-spanning network (Figure 1B) suggest that East Africa could represent the geographic origin of giraffes, consistent with the fact that the earliest fossil remains of *Giraffa camelopardalis *have been recovered in East Africa [21].

### Analysis of microsatellite loci

**Figure 2:**
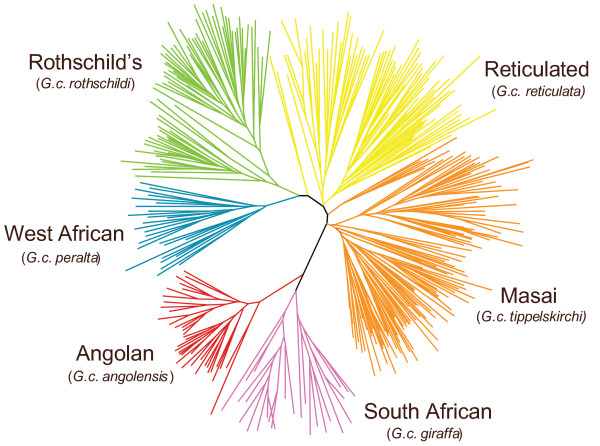
**Genetic subdivision in the giraffe based on microsatellites alleles**. Neighbor-joining network of allele-sharing distances (D_s_) based on 14 microsatellite loci typed in 381 giraffes. Colors are coded as in Figure 1A.

**Figure 3:**
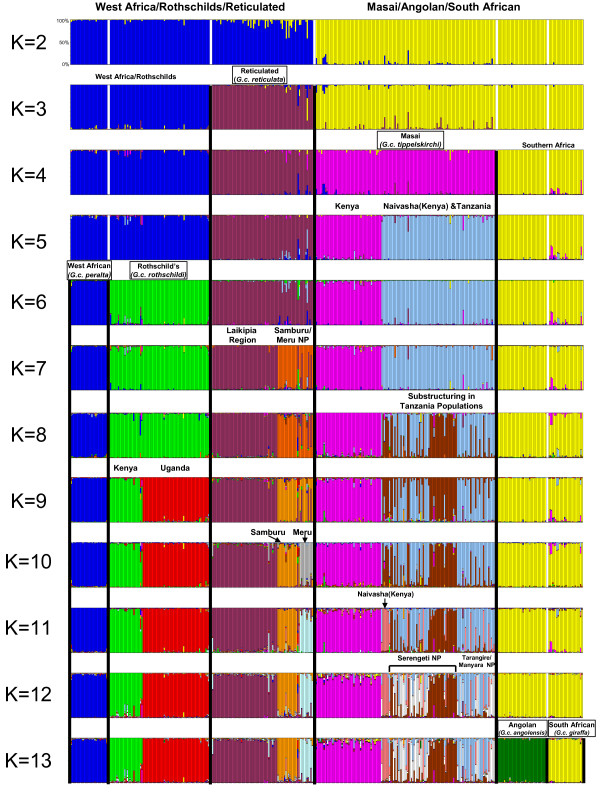
**Genetic subdivision among giraffe groups and populations based on Bayesian cluster analysis [23] of 14 microsatellite loci from 381 individuals**. Shown are the proportions of individual multilocus genotypes attributable to clusters (K) indicated by different colors. Sample group designations and sampling locations are denoted. We varied K from 2–16 and at least six groups corresponding to currently defined subspecies and 11 geographic clusters are resolved as indicated.

Genetic structure was also inferred for 381 individuals from 18 localities (see Additional file 10) using 14 unlinked microsatellite loci [22] all in Hardy-Weinberg equilibrium (see Additional files 11 and 12). Giraffe genotypes were strongly clustered into subspecific groups based on neighbor-joining analysis of allele-sharing genetic distances (Figure 2). Likewise, Bayesian clustering analysis of multilocus genotypes using STRUCTURE [23] resolved all six groups and, in addition, revealed striking subdivision at the population level, with 11 of the 18 sampling localities resolved as distinct genetic clusters at K = 13 and in assignment tests (Figure 3 and Additional file 13). Assignment probabilities correctly classified 371 of 381 (97%) individuals to population of origin (p > 0.90; see Additional file 13). Only three individuals were identified as hybrids between adjacent groups (see Additional files 14 and 15). Allele frequency differentiation of groups, as measured by F_st_, was significant for all pairwise comparisons (p < 0.05, G test) and ranged from 0.113 to 0.466 (see Additional file 16A). These results suggest low levels of gene flow among groups. Bayesian inference of migration rates using microsatellite data suggests migration rates are less than 0.2% per generation between the six subspecific groups (Additional file 17). The F_st _values among giraffe subspecies are comparable to that observed between forest and savannah species of the African elephant [6] and the levels of genetic structure observed within giraffe subspecies (Figures 2 and 3) are unprecedented for such a large and highly mobile African mammal.

Although hybridization in the wild has been reported for some subspecies (e.g., Masai and reticulated giraffes [2]), our results show little evidence for genetic exchange between subspecies or the 11 genetic units defined by STRUCTURE (Figures 2 and 3; Additional file 13). Only three potential subspecies hybrids in our sample of 381 individuals were identified by assignment tests (see Additional file 14) and include two Rothschild's/reticulated hybrids, and one Masai/reticulated hybrid. In fact, neighboring populations often show high levels of differentiation despite being closely situated (e.g., Masai, reticulated and Rothschild's giraffes in Kenya [2]) and not separated by apparent habitat or topographic boundaries. Within subspecies, the three reticulated giraffe localities were all significantly differentiated (microsatellite F_st _= 0.093–0.113; see Additional file 16D) even though they range from only 67 to 134 km distant. Genetic differentiation was also significant between four of the Masai giraffe sampling locations within Serengeti National Park (microsatellite F_st _= 0.080–0.126; see Additional file 16G) even through they range from only 60 to 130 km distant. The absence of genetic exchange, even between subspecies sharing a common geographic boundary, suggests they might be reproductively isolated.

## Discussion

### Historical causes of diversification

Our genetic results reveal the existence of sharp geographic subdivisions in mitochondrial DNA sequences and microsatellite allele frequencies, which are concordant with subspecific geographic ranges (Figures 1 and 2). Estimated divergence times among giraffe clades suggest a mid to late Pleistocene diversification of giraffes during a time of intense climatic change in sub-Saharan Africa [24-26]. Specifically, three climate-related factors could have influenced isolation among giraffe populations. First, paleoclimatic evidence indicates increasing aridity and cooler conditions beginning in the Late Pliocene that likely reduced connectivity between habitats favored by giraffes [24,27]. Second, pronounced periodic oscillations of wet and dry conditions driven by changes in the intensity and location of maximal insolation and with a 21000 year periodicity could have facilitated habitat fragmentation and population isolation [27,28]. Third, regional changes in habitat distribution could have promoted the isolation of specific populations, such as the expansion of the Mega Kalahari desert basin during dry periods of the Late Pleistocene that might have isolated Angolan and South African giraffe populations [29].

If climatic fluctuations caused broad scale changes in vegetation and habitat, leading to population divergence, then the phylogeographical patterns of giraffes should also be found in other species. Indeed, western, eastern, and southern African phylogeographic groupings in the giraffe mtDNA genealogy are broadly concordant with the genetic patterns observed in other taxa of large African mammals and might correspond to former habitat refuges [30]. For example, the phylogeographic patterns in the giraffe are strikingly congruent with those observed in the hartebeest (*Alcelaphus *spp.) complex [31,32]. Such concordance in phylogeographic patterns among multiple unrelated species suggests histories shaped by similar environmental forces, namely, changes in habitat related to climatic fluctuations [24], in agreement with models of environmentally driven evolution [33-35].

### Factors currently maintaining isolation among giraffe populations

Past historical factors leading to allopatric divergence could explain broad scale geographic divisions among western, eastern, and southern phylogeographic groupings. However, based on current knowledge of ecological or topographic factors, our results imply that those factors alone cannot explain the reproductive isolation among the parapatric subspecies of giraffes found in East Africa (Masai, Rothschild's, and reticulated giraffes). Indeed, despite suggestions that hybridization occurs frequently among giraffe subspecies, our microsatellite data suggests that such events are quite rare, occurring in only three of 381 sampled individuals (0.8%). In East Africa, one contributing factor to reproductive isolation might be natural selection for a reproductive cycle coincident with the emergence of new browse in contrasting climate regimes. The dry season occurs in July and August in the Sahelian zone north of the equator in contrast to December to March to the south [36]. Giraffes exhibit strong seasonality in births associated with the dry season when, in anticipation of the wetter conditions to follow, a flush of new tree shoots grow, and the protein content of browse is highest [37]. Rapid growth in juvenile giraffes is advantageous, especially during the first year, when mortality due to predation can range between 50–70% [12,38]. Consequently, hybridization between giraffes North and South of the equator could result in offspring born at the inappropriate season and have reduced fitness. Additionally, the apparent absence of strong post-mating barriers to reproduction in captivity [11,39] suggests a role for behavioral isolation in the wild. Our finding that pelage and genetic divergence in both maternally and biparentally inherited genetic markers are concordant on a broad geographic scale implicates pelage-based mate preferences as a possible isolating factor. Communication among giraffes is primarily visual [11] and given marked variation in pelage, assortative mating by pelage type could occur among some populations. For example, sexual imprinting, in which early exposure to conspecifics influences mate choice later in life [40], can cause speciation between populations that have diverged in allopatry and experience reinforcement upon secondary contact [41-43]. Although giraffes live in loose, non-territorial herds, calves are likely to be in close contact with only their mothers and other members of the local crèche until they are several months old [44]. Thus, individual giraffes might develop pelage-specific mate preferences, even in regions of overlap between subspecies. In zoo settings, visual barriers to interbreeding might not be maintained if calves are exposed to multiple pelage types. These tentative hypotheses need testing from comparative field studies focused on giraffe dispersal and reproductive biology.

The fine-scaled patterns of population isolation we observe within giraffe subspecies (Figure 3) are surprising given that giraffe social groupings and home ranges are known to be highly transient [2,4]. However, our genetic results suggest that giraffe social structure might be much more stable over the long term than has been shown by previous short-term observational field studies [4] and might also have a major influence on genetic differentiation among populations at larger geographic scales (i.e., subspecies).

### Genetic units and potential giraffe species

The concordant genetic and phenotypic divisions among giraffe subspecies (Figures 1 and 2) and the near absence of hybrids even between parapatric subspecies (e.g. among Masai, reticulated and Rothschild's giraffes) suggest that the giraffe might represent more than one species. Criteria for species designation vary according to the taxonomic group considered and the biological properties emphasized. For example, the biological species concept emphasizes reproductive isolation [1] whereas the phylogenetic species concept emphasizes fixed and diagnosable differences among species [45]. However, despite the differences among species concepts, there is a general consensus that species are separately-evolving metapopulation lineages united by gene flow [46]. We have shown that among giraffe subspecific groupings, including those that are adjacent to one another, there is an almost complete lack of gene flow, suggesting that these groups are reproductively isolated and thus constitute separate lineages. This conclusion is further supported by our mtDNA-based coalescence analysis, which indicates that groups have been isolated from one another between 0.13 and 1.62 MY (Table 2). The demonstration of largely independent gene pools using both organellar and nuclear loci among proximate populations constitutes one of the strongest forms of evidence that speciation has occurred [47]. Consequently, these arguments support viewing the giraffe as containing multiple distinct species rather than a single polytypic form. Reciprocal monophyly in mtDNA sequences and nearly absolute partitioning in microsatellite data support minimally six species, corresponding to *Giraffa peralta*, *G. rothschildi*, *G. reticulata*, *G. tippelskirchi*, *G. giraffa*, and *G. angolensis*. Further, the Masai giraffe might constitute more than one species, consistent with its subdivision into populations east and west of the Rift Valley. However, additional taxa might be defined, pending analysis of the subspecies included in taxonomic schemes (Table 1) not sampled by our study design (e.g., *G. c. antiquorum*[10]). Finally, many of these species appear to include multiple distinct population units that are genetically differentiated. Despite the demonstrated capability for long distance dispersal [3], ecological, historical and behavioral factors could have collectively caused differentiation in the giraffe comparable to that of highly sedentary species. Consequently, individual dispersal, even in one of the most highly vagile terrestrial vertebrates, does not preclude an as yet uncertain isolating mechanism.

## Conclusion

We have shown that, despite a high capacity for dispersal, the giraffe exhibits extensive population genetic structure in both mitochondrial and nuclear DNA markers. Further, our results indicate that neighbouring subspecies as well as those that are geographically separated are essentially reproductively isolated, suggesting that some might represent distinct species rather than a single polytypic form. Minimally, the seven lineages that are reciprocally monophyletic in the mtDNA tree (Figure 1A) need to be considered evolutionarily significant units if not species, even under the most conservative definition of the term, whereas the remaining populations should be considered independent genetic units [48,49], all needing separate population management.

Our results have important implications with regards to the conservation of giraffe populations. Giraffes were once continuously distributed throughout the dry savannas of Africa, from Algeria and Morocco to South Africa [2,11]. However, the geographic range of the giraffe has been severely fragmented during historic times due to increasing aridity and human population growth so that today, giraffes are discontinuously distributed from the Sahel to South Africa. Recent estimates indicate that approximately 110000 giraffes exist in Africa [13], but many populations are declining due to human pressures. For example, severe poaching and armed conflict in Somalia, Ethiopia, and Kenya reduced the number of reticulated giraffes (*G.c. reticulata*) from about 27000 individuals in the 1990s to currently fewer than 3000 individuals ([13]; N. Georgiadis, unpublished results). The evolutionarily significant units that we have uncovered and their isolated constituent populations merit conservation and separate management. Several of these previously unrecognized genetic units are highly endangered, such as the West African giraffe, numbering about only 100 individuals and restricted to a single area in Niger [13,50]. However, the giraffe is currently listed as Lower Risk by the IUCN Red List [51] under the assumption that all giraffe populations are considered a single species and therefore managed as such. Our results show that even within well known and highly mobile species, subdivisions can exist and their lack of recognition could lead to further endangerment or even extinction [52].

## Methods

### Sample collection

Skin biopsies were taken by remote system delivery of biopsy darts. We used a CO_2 _powered DanInject (Børkop, Denmark) Model JM with both in house and manufactured (Palmer Capshur, Atlanta, GA, USA) 6 mm biopsy darts. Where observed, we attempted to sample distinct groups within each subspecies. The presence of so many mtDNA haplotypes within many subspecies suggests multiple matralines were sampled (see results). Subspecies assignments for each sampled giraffe were based on geographic location and pelage following Dagg and Foster [11]. The skin samples were placed in 0.5 ml room temperature tissue preservative buffer for preservation. Samples were transferred to the same buffer but with 0.2% gluteraldehyde for sterilization before export/import to the USA. The sampling performed by HDZ researchers was performed under Kenyan permit KE911780-1, Ugandan permits UWA/PMR/RES/50 and Ugandan National Council for Science and Technology permit #EC549, Niger Interior Ministry Permit 731 and Namibian Ministry of Environment and Tourism Research/Collection Permit #597/2002. All samples were imported under USDA/APHIS Import Permit #43686. Detailed permit information is available on request to the authors. We extracted genomic DNA from giraffe biopsy samples using a standard phenol chloroform/isoamyl alcohol extraction protocol.

### Mitochondrial DNA

A 654-nucleotide fragment of mtDNA was amplified and sequenced in 266 giraffes and one okapi (*Okapia johnstoni*). We amplified and sequenced this fragment using the primers L15774 and H16498 [53]. Polymerase chain reaction amplification was performed in a 50 μl reaction using an MWG-Biotech Primus 96 Plus thermal cycler with 35.7 μl sterile double-distilled water, 5 μl 10 × PCR buffer, 5 μl of 25 mM MgCl_2_, 1 μl of 10 mM dNTP mix, 1 μl of both 25 pM/μl forward and reverse primers, 0.3 μl *Taq *polymerase (Sigma-Aldrich, St Louis, MO, USA), and approximately 50 ng of genomic DNA. The PCR amplification profile was 94°C for 3 min, followed by 30 cycles of 94°C for 30 s, a primer-specific annealing temperature for 35 s, 72°C for 45 s, ending with a single extension of 72°C for 5 min. All PCRs included a negative control (no DNA). PCR products of expected size were excised from 1% agarose/Tris/acetic acid/EDTA gels and purified using an Ultra Clean Kit (MoBio Laboratories, Solana Beach, CA, USA). The mitochondrial fragment was sequenced in both forward and reverse directions on a Beckman CEQXL2000 capillary sequencer (Beckman Coulter, Fullerton, CA, USA). Sequences were aligned using Sequencher 3.0 (Gene Codes Corp., Ann Arbor, MI, USA).

The mtDNA data matrix (n = 266 sequences) was collapsed to 35 haplotypes using the program Collapse v1.1 [54]. To ensure proper phylogenetic resolution and robust support for relationships among haplotypes, the rest of the *CYTb *gene was amplified and sequenced from 35 giraffes, representing each of the 35 unique haplotypes, and the okapi. Primers L14724 [55], L15162, and H15915 [56] were used to amplify and sequence the *CYTb *gene using the same protocols described above. This sequence was then concatenated with the 654 nt fragment, resulting in an alignment length of 1709 nt (with okapi) or 1707 nt (without okapi). These sequences were deposited in GenBank (accession numbers EU088317–EU88352).

Phylogenetic relationships among the 35 giraffe haplotypes (1709 nt) were estimated using maximum parsimony (equally weighted) (MP), maximum likelihood (ML), and minimum evolution (ME) methods. The HKY85+I+G model of DNA substitution was selected [57] and used in ML and ME analyses that included only giraffe haplotypes. For analyses that included the okapi, the HKY85 (without accounting for site heterogeneity) model was used. Gaps (insertions/deletions) were coded as a fifth base in MP analyses. Maximum parsimony and ME analyses were executed in PAUP* 4.0b10 [58]. For these analyses, heuristic searches were performed using 100 random sequence additions, with one tree held at each step during stepwise addition, tree-bisection-reconnection branch swapping, steepest descent option not in effect, no upper bound for MaxTrees, and MulTrees option in effect. Maximum likelihood analyses were conducted with TREEFINDER [59] and parameters of the HKY85+I+G or HKY85 model were estimated along with the tree topology. For each phylogenetic method, robustness of clades was assessed using 1000 bootstrap pseudoreplicates. The okapi sequence was used to root the phylogenies of the giraffe haplotypes. However, due to the large sequence divergence between giraffe and okapi (and therefore the potential for signal saturation), phylogenetic trees including only giraffe haplotypes were also midpoint rooted. Regardless of rooting method or optimality criterion used, clades with a ≥ 80% bootstrap value were maintained across all analyses (Figure 1A and Additional files 2, 3, 4, 5, 6, 7).

### Minimum-spanning network between haplotypes

We also constructed a minimum-spanning network of absolute distances between control region haplotypes using the molecular-variance parsimony algorithm as implemented in Arlequin v3.1 [20].

### Genetic structure and diversity

Population structure was deduced with an analysis of molecular variance (AMOVA) using Arlequin V3.1 [20]. In order to identify groups of populations based on genetic differences, we grouped sampling localities to maximise the among-group variance component (Φct). Haplotype and nucleotide diversity indices were calculated with Arlequin V3.1 using mtDNA control region data.

### Estimation of divergence times using MDIV [19]

We generated maximum likelihood estimates of θ, twice the effective female population size (N_*fe*_) times the mutation rate (*u*); *T*, the divergence time between two populations scaled by population size; and *M*, the gene migration rate between the two populations, also scaled by population size. We assumed uniform prior distributions and applied an HKY model of mutation [60] to allow for the possibility of multiple mutations per site. We ran Markov chains of 4000000 cycles preceded by a "burn-in" period of 500000 cycles for each pairwise population comparison, set maximum values for *T *and *M *of 10 and 30, respectively, and ran the analysis three times for each population comparison using different random seeds. We calculated divergence time (*t*) using the formula *t *= T *θ*/*(*2u*)*g, where T and θ are generated by the program, *u *is the mutation rate, and *g *is generation time in years. We calculated *u *as 2*μ*k, where *μ *is the mutation rate per nucleotide and *k *is the length of the sequence. Given the higher mutation rate found in the control region relative to the cytochrome *b *gene, we used a range of estimated mutation rates for control region sequence that span values found previously in other large mammal species. These included 0.05, 0.10 and 0.15 substitutions/site/MY, and a generation time of 4 years, which is the approximate age of first breeding for giraffes [38].

### Microsatellite amplification/genotyping

We amplified 13 published [22] and one novel (see Additional file 18) giraffe-specific microsatellite loci to generate multilocus genotypes for the 381 individuals. We performed the PCR amplification in 25 μl reaction volumes using an ABI 480 thermocycler (Perkin-Elmer; Foster City, CA, USA) with approximately 50 ng of genomic DNA as template. Final amplification conditions consisted of 12.5 pmol unlabelled reverse primer, 12.5 pmol fluorescently labeled forward primer, 1.5 mM MgCl_2_, 200 μM each dNTP, and 0.5 units of *Taq *DNA polymerase (Promega; Madison, WI, USA). The thermal profile for PCR amplification was 95°C for 5 min, followed by 35 cycles of denaturing at 95°C for 30 s, a annealing at primer-specific temperature for 30 s, and elongated at 72°C for 30 s, ending with a single extension of 72°C for 10 min. We separated the PCR products on either a 7% polyacrylamide gel electrophoresed on an ABI 377 or through POP4 capillary buffer electrophoresed on an ABI 3100 DNA Analyzer (Applied Biosystems, Inc; Foster City, CA, USA). We assigned allele fragment lengths relative to the GeneScan-500 (TAMRA; Applied Biosystems, Inc; Foster City, CA, USA) size standard using the ABI GeneScan software program. We checked and corrected the data set for errors using MICRO-CHECKER 2.2.3 [61] and MSA 4.00 [62].

### Microsatellite analysis

Allelic diversity, and expected (H_e_) and observed (H_o_) heterozygosity were calculated using the program GENALEX [63]. Each locus was tested for deviation from Hardy-Weinberg equilibrium and linkage disequilibrium with other loci (p < 0.05) using the program Genepop [64]. Bonferroni corrections to significance values [65] were applied to account for multiple tests (see Additional files 12A and 12B).

### Allele-sharing neighbour-joining network

We generated the neighbor-joining network tree using 14-locus genotypes of 381 individuals. The network was created using the allele-sharing distance D_s _[66] and the program POPULATIONS v1.2.28 [67].

### Bayesian clustering analysis

We used the program STRUCTURE [23] to infer genetic population structure using genotypes from 14 microsatellite loci of 381 individuals. All individuals were combined into one dataset for analysis, without any *a priori *population assignments and admixture was allowed. We evaluated K values, the number of assumed populations, from 1–16 using a burn-in of 50000 iterations followed by 500000 iterations for each value of K. Each value of K was run a minimum of three times to evaluate stability (see Additional file 19). We then calculated the posterior probability of population assignment to one of the six subspecies using initial assignments based on the *a priori *K = 13 cluster proportion results, with the migration parameter set to γ = 0.1 (see Additional file 13A). We also utilized the program GENECLASS2 [68] for a comparative estimate of population assignments using the same K = 13 cluster proportion results for initial population assignments. We used the Rannala and Mountain [69] Bayesian assignment method with the simulation method of Paetkau [70] and an assignment threshold level of 0.05 (see Additional file 13B).

### Population differentiation and inbreeding coefficients

We calculated pairwise F_st _values using microsatellite results for population comparisons at the subspecies, population and sampling site levels using the program Fstat [71] (see Additional file 16A). Significant values of Fst were determined using the G test in Fstat (α = 0.05). We also calculated Nei's genetic distance [72] for population comparisons at the subspecies level (GENALEX [63]) (see Additional file 16B). Population inbreeding coefficients (Fis) were also calculated using Fstat and significant values determined using α = 0.05 (see Additional file 20).

### Migration-rate estimation

Recent migration rates between the six giraffe subspecies were estimated using a Bayesian MCMC analysis of microsatellite genotypes (BayesAss 1.3 [73]). Individuals were preassigned to the six subspecies based on sampling location. We used 3000000 iterations, a sampling frequency of 2000, a burn-in length of 999999 iterations, and delta values for allele frequency, migration rate and level of inbreeding of 0.15 (see Additional file 17).

### Isolation by distance

We tested for isolation by distance between subspecies, populations and sample locations using a comparison of genetic distance (F_st_/(1-F_st_)) with geographic distance, applying the Mantel test in GENALEX [63] (999 permutations, significance level p < 0.01) (see Additional file 21).

### Molecular analysis of variance – microsatellites

We calculated molecular analysis of variance (AMOVA) for microsatellite data at the subspecies (Q = 6) and population (Q = 10) levels (999 permutations) using the program GENALEX [63] (see Additional file 22).

## Authors' contributions

DMB conceived and designed the study and collected the mitochondrial sequence data and contributed to writing the paper. RAB and EEL helped in study design, obtained giraffe samples, and collected the microsatellite data. NJG helped in study design and obtained giraffe samples. KPK helped collect additional mitochondrial sequence data, performed the phylogenetic analyses and contributed to writing the paper. JPP analyzed the microsatellite data and contributed to writing the paper. BM performed the population genetic analyses and divergence dating analyses on the mitochondrial sequence data and contributed to writing the paper. GFG contributed to writing the paper. DKJ contributed to writing the paper. RKW supervised the study and contributed to writing the paper. All authors read and approved the final manuscript.

## Supplementary Material

Additional file 1 Table showing giraffe sampling localities and sample sizes for mtDNA characterization with resulting mtDNA control region haplotypes Click here for file

Additional file 2 Figure showing maximum parsimony phylogeny of giraffe (*Giraffa camelopardalis*) mtDNA haplotypes, rooted with okapi (*Okapia johnstoni*) Click here for file

Additional file 3 Figure showing maximum parsimony phylogeny of giraffe (*Giraffa camelopardalis*) mtDNA haplotypes, rooted using midpoint rooting Click here for file

Additional file 4 Figure showing minimum evolution phylogeny of giraffe (*Giraffa camelopardalis*) mtDNA haplotypes, rooted with okapi (*Okapia johnstoni*) Click here for file

Additional file 5 Figure showing minimum evolution phylogeny of giraffe (*Giraffa camelopardalis*) mtDNA haplotypes, rooted using midpoint rooting Click here for file

Additional file 6 Figure showing maximum likelihood phylogeny of giraffe (*Giraffa camelopardalis*) mtDNA haplotypes, rooted with okapi (*Okapia johnstoni*) Click here for file

Additional file 7 Figure showing maximum likelihood phylogeny of giraffe (*Giraffa camelopardalis*) mtDNA haplotypes, rooted using midpoint rooting Click here for file

Additional file 8 Table of AMOVA results according to subspecific groupings Click here for file

Additional file 9 Table of sample sizes, number of mitochondrial haplotypes and molecular diversity indices per sampling locality and subspecies Click here for file

Additional file 10 Table of sampling locations (six historical subspecies, 30 sample sites, 381 individuals) for microsatellite characterization Click here for file

Additional file 11 Table of summary statistics for microsatellite data (381 specimens, all populations and pelage subspecies) Click here for file

Additional file 12 Tables showing (A) observed and expected heterozygosity, and deviations from Hardy-Weinberg equilibrium in six giraffe subspecific populations, and (B) observed and expected heterozygosity, and deviations from Hardy-Weinberg equilibrium in 16 giraffe populations Click here for file

Additional file 13 Tables showing (A) posterior probability population assignments of 381 Individuals, based on assignment to pelage/subspecies designations using STRUCTURE [19], and (B) subspecies assignment of 381 individuals, based on assignment to pelage/subspecies designations, using multilocus genotypes and Bayesian analysis (Rannala and Mountain method in Geneclass2 [46,47]) Click here for file

Additional file 14 Table of STRUCTURE [23] cluster results identify three possible subspecies hybrids, four population hybrids within the same subspecies and one possible population migrant within the same subspecies Click here for file

Additional file 15 Figure showing STRUCTURE [19] cluster assignments of detected giraffe hybrids Click here for file

Additional file 16 Tables showing (A) pairwise F_st _values and statistical significance for the six giraffe subspecies; (B) pairwise values for Nei's genetic distance among six giraffe subspecies; (C) *G.c. angolensis *(Angolan) population pairwise comparison Fst values and statistical significance; (D) *G.c. reticulata *(Reticulated) population pairwise comparison Fst values and statistical significance; (E) *G.c. rothschildi *(Rothschild's) population pairwise comparison Fst values and statistical significance; (F) *G.c. tippelskirchi *(Masai) population pairwise comparison Fst values and statistical significance; and (G) *G.c. tippelskirchi *(Masai) Serengeti N.P. population pairwise comparison Fst values and statistical significance Click here for file

Additional file 17 Table showing migration rates among giraffe subspecies Click here for file

Additional file 18 Table of primer sequences and amplification characteristics of *Giraffa camelopardalis *microsatellite locus NECK484 Click here for file

Additional file 19 Figure showing likelihood values for inferred number of genetic clusters (K) from STRUCTURE [23] (three iterations per value of K) Click here for file

Additional file 20 Table of overall F_is _values per subspecies, and per population Click here for file

Additional file 21 Table of Mantel test of isolation by distance results (correlation of genetic distance (Fst/(1-Fst) with geographic distance) Click here for file

Additional file 22 Table of AMOVA using microsatellite data results Click here for file
