# Supplementary material for: Extensive population genetic structure in the giraffe
Source: BMC Biol. 2007 Dec 21;5:57. doi: 10.1186/1741-7007-5-57 (PMC2254591; doi:10.1186/1741-7007-5-57)
Supplement: Additional file 1 — Table showing giraffe sampling localities and sample sizes for mtDNA characterization with resulting mtDNA control region haplotypes [file 1741-7007-5-57-S1.DOC]

**Additional file 1.** Giraffe sampling localities and sample sizes for mtDNA characterization with resulting mtDNA control region haplotypes.

| Subspecies | **Population/Site** | **n** | **Haplotypes**  **(frequency)** |
| --- | --- | --- | --- |
| **G.c. peralta** **(West African)** | Harikanassou Region, Niger | **22** | 7(7), 8(15) |
| ***G.c. rothschildi* (Rothschild’s)** |  | **51** |  |
|  | Murchison Falls N. P., Uganda | 25 | 9(25) |
|  | Nakuru N. P., Kenya | 14 | 9(7), 10(6), 11(1) |
|  | Ruma, Kenya | 12 | 9(9), 10(2), 11(1) |
| ***G.c. reticulata***  **(Reticulated)** |  | **63** |  |
|  | Ol Jogi, Laikipia, Kenya | 32 | 12(2), 13(12), 14(6), 15(5), 25(1), 26(6) |
|  | Sweetwaters, Laikipia, Kenya | 10 | 13(1), 14(2), 15(6), 34(1) |
|  | Meru N. P., Kenya | 6 | 13(5), 15(1) |
|  | Samburu N. R., Kenya | 15 | 13(2), 14(7), 15(1), 26(5) |
| ***G.c. tippelskirchi***  **(Masai)** |  | **83** |  |
|  | Athi River Ranch, Kenya | 17 | 1(2), 2(9), 3(5), 4(1) |
|  | Chyulu Hills, Kenya | 16 | 1(2), 3(13), 16(1) |
|  | Lobo, Serengeti N. P., Tanzania | 5 | 18(5) |
|  | Ndutu, Serengeti N. P., Tanzania | 11 | 18(7), 20(2), 21(2) |
|  | Ngorongoro, Serengeti N. P., Tanzania | 1 | 20(1) |
|  | Seronera, Serengeti N. P., Tanzania | 4 | 18(4) |
|  | Varicho, Serengeti N. P., Tanzania | 1 | 23(1) |
|  | Manyara N. P., Tanzania | 9 | 1(2), 3(5), 18(2) |
|  | Lake Naivasha, Tanzania | 6 | 1(3), 3(3) |
|  | Tarangire N. P., Tanzania | 13 | 1(1), 3(7), 22(2), 23(1), 24(2) |
| **G.c. angolensis** **(Angolan)** |  | **35** |  |
|  | Etosha N. P., Namibia | 15 | 27(1), 28(2), 29(2), 30(10) |
|  | Hoanib River, Namibia | 13 | 33(13) |
|  | Hoarsib River, Namibia | 2 | 31(1), 32(1) |
|  | Kamanjab, Namibia | 1 | 27(1) |
|  | Khumib River, Namibia | 4 | 33(3) |
| **G.c. giraffa** **(South African)** |  | **12** |  |
|  | Kruger N. P., South Africa | 11 | 5(10), 6(1) |
|  | Serondella, Zimbabwe | 1 | 5(1) |
