# Supplementary material for: Extensive population genetic structure in the giraffe
Source: BMC Biol. 2007 Dec 21;5:57. doi: 10.1186/1741-7007-5-57 (PMC2254591; doi:10.1186/1741-7007-5-57)
Supplement: Additional file 3 — Figure showing maximum parsimony phylogeny of giraffe (Giraffa camelopardalis) mtDNA haplotypes, rooted using midpoint rooting [file 1741-7007-5-57-S3.DOC]

**Additional file 3.** Maximum parsimony phylogeny of giraffe (*Giraffa camelopardalis*) mtDNA haplotypes. The phylogeny is a strict consensus of 45 equally-parsimonious trees and midpoint rooted. Tree length = 216, consistency index (excluding uninformative characters) = 0.7049, retention index = 0.9445, and 128 parsimony-informative nucleotide changes. Bootstrap values ≥50%, based on 1000 pseudoreplicates, are shown above internodes.
