# Supplementary material for: Extensive population genetic structure in the giraffe
Source: BMC Biol. 2007 Dec 21;5:57. doi: 10.1186/1741-7007-5-57 (PMC2254591; doi:10.1186/1741-7007-5-57)
Supplement: Additional file 4 — Figure showing minimum evolution phylogeny of giraffe (Giraffa camelopardalis) mtDNA haplotypes, rooted with okapi (Okapia johnstoni) [file 1741-7007-5-57-S4.DOC]

**Additional file 4.** Minimum evolution phylogeny of giraffe (*Giraffa camelopardalis*) mtDNA haplotypes, based on HKY85 corrected distances and rooted with *Okapia johnstoni*. Minimum evolution score = 0.2696. Bootstrap values ≥50%, based on 1000 pseudoreplicates, are shown above internodes. Terminal names refer to haplotype numbers and subspecies group. Branch lengths are proportional to number of substitutions per site (scale bar).
