# Supplementary material for: Extensive population genetic structure in the giraffe
Source: BMC Biol. 2007 Dec 21;5:57. doi: 10.1186/1741-7007-5-57 (PMC2254591; doi:10.1186/1741-7007-5-57)
Supplement: Additional file 5 — Figure showing minimum evolution phylogeny of giraffe (Giraffa camelopardalis) mtDNA haplotypes, rooted using midpoint rooting [file 1741-7007-5-57-S5.DOC]

**Additional file 5.** Minimum evolution phylogeny of giraffe (*Giraffa camelopardalis*) mtDNA haplotypes, based on HKY85+I+Γ corrected distances. The tree is midpoint rooted. Minimum evolution score = 0.2696. Bootstrap values ≥50%, based on 1000 pseudoreplicates, are shown above internodes. Branch lengths are proportional to number of substitutions per site (scale bar).
