# Supplementary material for: Extensive population genetic structure in the giraffe
Source: BMC Biol. 2007 Dec 21;5:57. doi: 10.1186/1741-7007-5-57 (PMC2254591; doi:10.1186/1741-7007-5-57)
Supplement: Additional file 6 — Figure showing maximum likelihood phylogeny of giraffe (Giraffa camelopardalis) mtDNA haplotypes, rooted with okapi (Okapia johnstoni) [file 1741-7007-5-57-S6.DOC]

**Additional file 6.** Maximum likelihood phylogeny of giraffe (*Giraffa camelopardalis*) mtDNA haplotypes, using the HKY85 model and rooted with *Okapia johnstoni*. –Ln likelihood = 4483.9209, TS/TV ratio = 10.395, A = 0.32602, C = 0.27754, G = 0.13207, T = 0.26437. Bootstrap values ≥50%, based on 1000 pseudoreplicates, are shown above internodes. Terminal names refer to haplotype numbers and subspecies group. Branch lengths are proportional to number of substitutions per site (scale bar).
