# Supplementary material for: Extensive population genetic structure in the giraffe
Source: BMC Biol. 2007 Dec 21;5:57. doi: 10.1186/1741-7007-5-57 (PMC2254591; doi:10.1186/1741-7007-5-57)
Supplement: Additional file 7 — Figure showing maximum likelihood phylogeny of giraffe (Giraffa camelopardalis) mtDNA haplotypes, rooted using midpoint rooting [file 1741-7007-5-57-S7.DOC]

**Additional file 7.** Maximum likelihood phylogeny of giraffe (*Giraffa camelopardalis*) mtDNA haplotypes, using the HKY85+I+Γ model. The tree is midpoint rooted and is the same tree shown in Fig. 1a. . –Ln likelihood = 3624.1123, TS/TV ratio = 58.3510, I = 0.4592, Γ = 0.2411, A = 0.32610, C = 0.27693, G = 0.13196, T = 0.26502. Bootstrap values ≥50%, based on 1000 pseudoreplicates, are shown above internodes. Branch lengths are proportional to number of substitutions per site (scale bar).
