# Supplementary material for: Extensive population genetic structure in the giraffe
Source: BMC Biol. 2007 Dec 21;5:57. doi: 10.1186/1741-7007-5-57 (PMC2254591; doi:10.1186/1741-7007-5-57)
Supplement: Additional file 8 — Table of AMOVA results according to subspecific groupings [file 1741-7007-5-57-S8.DOC]

Additional file 8. AMOVA results according to subspecific groupings. AMOVA uses the frequencies of haplotypes and the number of mutations among them to test the significance of the variance components associated with various hierarchical levels of genetic structure (within populations, among populations within groups, and among groups) by means of non-parametric permutation methods. In order to identify groups of populations based on genetic differences, we grouped sampling localities in a way that maximised the among-group variance component (Φ_ct_). Differentiation between groups was tested with an exact test of population differentiation using 10,000 Markov chain steps in arlequin 3.1. per = *peralta*, rot = *rothschildi*, ret = *reticulata*, tip = *tippleskirschi*, ang = *angolensis*, gir = *giraffa*.

| Grouping tested | df | SS | Variance component | % of variance |
| --- | --- | --- | --- | --- |
| [per][rot][ret][tip][gir][ang] |  |  |  |  |
| Among Groups [Ф_ct_] | 5 | 1833.74 | 8.255 | 75.37*** |
| Among Populations [Ф_sc_] | 19 | 249.06 | 1.318 | 12.03*** |
| Within Populations [Ф_st_] | 241 | 332.45 | 1.379 | 12.59*** |
| [per][rot+ret+tip][gir+ang] |  |  |  |  |
| Among Groups [Ф_ct_] | 2 | 601.10 | 3.369 | 29.36*** |
| Among Populations [Ф_sc_] | 22 | 1480.81 | 6.728 | 58.62*** |
| Within Populations [Ф_st_] | 241 | 332.45 | 1.379 | 12.02*** |
| [per][rot+ret+tip][gir][ang] |  |  |  |  |
| Among Groups [Ф_ct_] | 3 | 800.10 | 4.625 | 38.16*** |
| Among Populations [Ф_sc_] | 21 | 1282.72 | 6.116 | 50.46*** |
| Within Populations [Ф_st_] | 241 | 332.45 | 1.379 | 11.38*** |
| [per+rot+ret+tip+gir][ang] |  |  |  |  |
| Among Groups [Ф_ct_] | 1 | 482.32 | 6.545 | 45.01*** |
| Among Populations [Ф_sc_] | 23 | 1600.49 | 6.618 | 45.51*** |
| Within Populations [Ф_st_] | 241 | 332.45 | 1.379 | 9.49*** |
| [per+rot+ret][tip+gir][ang] |  |  |  |  |
| Among Groups [Ф_ct_] | 2 | 1190.59 | 6.830 | 56.45*** |
| Among Populations [Ф_sc_] | 22 | 892.22 | 3.890 | 32.15*** |
| Within Populations [Ф_st_] | 241 | 332.45 | 1.379 | 11.40*** |
| [per+rot+ret+tip][gir+ang] |  |  |  |  |
| Among Groups [Ф_ct_] | 1 | 436.75 | 4.526 | 35.60*** |
| Among Populations [Ф_sc_] | 23 | 1646.05 | 6.809 | 53.55*** |
| Within Populations [Ф_st_] | 241 | 332.45 | 1.379 | 10.85*** |
