# Supplementary material for: Extensive population genetic structure in the giraffe
Source: BMC Biol. 2007 Dec 21;5:57. doi: 10.1186/1741-7007-5-57 (PMC2254591; doi:10.1186/1741-7007-5-57)
Supplement: Additional file 9 — Table of sample sizes, number of mitochondrial haplotypes and molecular diversity indices per sampling locality and subspecies [file 1741-7007-5-57-S9.DOC]

Additional file 9. Sample sizes, number of haplotypes and molecular diversity indices per sampling locality and subspecies.

| Subspecies | **Population/Site** | **n** | **#haps** | ***h*** | ***π*** |
| --- | --- | --- | --- | --- | --- |
| **G.c. peralta** |  | **22** |  | ***0.4545±0.0777*** | ***0.001062±0.001087*** |
|  | Niger | 22 | 2 | 0.4545±0.0777 | 0.001062±0.001087 |
| ***G.c. rothschildi*** |  | **51** |  | ***0.3341±0.0752*** | ***0.000810±0.000899*** |
|  | Uganda | 25 | 1 | 0 | 0 |
|  | Nakuru | 14 | 3 | 0.6044±0.0759 | 0.001566±0.001430 |
|  | Ruma | 12 | 3 | 0.4394±0.1581 | 0.001097±0.001154 |
| ***G.c. reticulata*** |  | **63** |  | ***0.7803±0.0213*** | ***0.015886±0.008410*** |
|  | Ol Jogi | 32 | 6 | 0.7843±0.0431 | 0.017862±0.009515 |
|  | Sweetwaters | 10 | 4 | 0.6444±0.1518 | 0.009212±0.005698 |
|  | Meru | 6 | 2 | 0.3333±0.2152 | 0.007806±0.005371 |
|  | Samburu | 15 | 4 | 0.6952±0.0815 | 0.009055±0.005400 |
| ***G.c. tippelskirchi*** |  | **83** |  | ***0.7743±0.0324*** | ***0.017644±0.009220*** |
|  | Athi River | 17 | 4 | 0.6544±0.0891 | 0.023109±0.012454 |
|  | Chyulu Hills | 16 | 3 | 0.3417±0.1403 | 0.001132±0.001152 |
|  | Lobo | 5 | 1 | 0 | 0 |
|  | Ndutu | 11 | 3 | 0.5818±0.1420 | 0.006132±0.004000 |
|  | Ngorongoro | 1 | 1 | 0 | 0 |
|  | Seronera | 4 | 1 | 0 | 0 |
|  | Varicho | 1 | 1 | 0 | 0 |
|  | Manyara | 9 | 3 | 0.6667±0.1318 | 0.009107±0.005718 |
|  | Lake Naivasha | 6 | 2 | 0.6000±0.1291 | 0.001405±0.001481 |
|  | Tarangire | 13 | 5 | 0.7051±0.1220 | 0.002222±0.001823 |
| ***G.c. angolensis*** |  | **35** |  | ***0.6908±0.0611*** | ***0.007634±0.004476*** |
|  | Etosha | 15 | 4 | 0.5524±0.1374 | 0.009212±0.005479 |
|  | Hoanib River | 13 | 1 | 0 | 0 |
|  | Hoarsib River | 2 | 2 | 1.0000±0.5000 | 0.011682±0.012797 |
|  | Kamanjab | 1 | 1 | 0 | 0 |
|  | Khumib River | 4 | 1 | 0 | 0 |
| **G.c. giraffa** |  | **12** |  | ***0.1818±0.1436*** | ***0.000426±0.000670*** |
|  | Kruger | 11 | 2 | 0.1818±0.1436 | 0.000426±0.000670 |
|  | Zimbabwe | 1 | 1 | 0 | 0 |
