# Supplementary material for: Extensive population genetic structure in the giraffe
Source: BMC Biol. 2007 Dec 21;5:57. doi: 10.1186/1741-7007-5-57 (PMC2254591; doi:10.1186/1741-7007-5-57)
Supplement: Additional file 10 — Table of sampling locations (six historical subspecies, 30 sample sites, 381 individuals) for microsatellite characterization [file 1741-7007-5-57-S10.DOC]

**Additional file 10.** Sampling locations (6 historical subspecies, 30 sample sites, 381 individuals) for microsatellite characterization.

| Subspecies | **Geographic Location** | **Population/Site** | **No. of Samples** | **Location**  **(Lat Long)** |
| --- | --- | --- | --- | --- |
| **G.c. peralta (West African)** | Niger | Niger | 28 | N16.00° E8.00° |
|  |  |  |  |  |
| ***G.c. rothschildi*** **(Rothschild’s)** | Uganda | Murchison Falls N.P. | 51 | N2.0833° E34.0833° |
|  | Kenya | Nakuru N.P. | 18 | N1° E35° |
|  |  | Ruma | 12 | N1° E35° |
|  |  |  |  |  |
| ***G.c. reticulata* (Reticulated)** | Kenya | Laikipia (Ol Jogi Pyramid) | 23 | N0.3125° E36.9925° |
|  |  | Laikipia (Ol Jogi Ranch) | 27 | N0.3125° E36.9925° |
|  |  | Laikipia (Sweetwaters) | 2 | N0.3125° E36.9925° |
|  |  | Meru N.P. | 10 | N0.2400° E38.1972° |
|  |  | Samburu N.R. | 18 | N0.6036° E37.5289° |
|  |  |  |  |  |
| ***G.c. tippelskirchi* (Masai)** | Kenya | Athi River Ranch | 25 | S1.5125° E37.0425° |
|  |  | Chyulu Hills | 25 | S1.5219° E37.7278° |
|  | Tanzania | Serengeti N.P. Grumeti | 4 | S1.9281° E35.0081° |
|  |  | Serengeti N.P. Kirawira | 7 | S2.0997° E34.1933° |
|  |  | Serengeti N.P. Kuka | 1 | S1.7444° E35.1878° |
|  |  | Serengeti N.P. Lobo | 1 | S2.0131° E35.2164° |
|  |  | Serengeti N.P. Magogwe | 1 | S1.7275° E35.2006° |
|  |  | Serengeti N.P. Musabi | 1 | S2.1636° E34.4986° |
|  |  | Serengeti N.P. Ndutu | 16 | S3.0025° E34.9386° |
|  |  | Serengeti N.P.Ngorogoro | 1 | S3.1400° E35.4336° |
|  |  | Serengeti N.P. Seronera | 19 | S2.4272° E34.9131° |
|  |  | Serengeti N.P. Varicho | 6 | S2.2436° E34.4117° |
|  |  | Manyara N.P. | 12 | S3.6389° E35.7075° |
|  |  | Lake Naivasha | 7 | S0.8458° E36.2250° |
|  |  | Tarangire N.P. | 17 | S3.9328° E36.0817° |
|  |  |  |  |  |
| **G.c. angolensis (Angolan)** | Namibia | Etosha National Park | 15 | S18.5858° E16.8884° |
|  |  | Hoanib River | 15 | S19.2421° E13.3717° |
|  |  | Hoarsib River | 3 | --- ---- |
|  |  | Kamanjab | 1 | --- ---- |
|  |  | Khumib River | 4 | S18.6636° E13.6387° |
|  |  |  |  |  |
| **G.c. giraffa (South African)** | South Africa | Kruger National Park | 27 | S23.8320° E31.5690° |
