# Supplementary material for: Extensive population genetic structure in the giraffe
Source: BMC Biol. 2007 Dec 21;5:57. doi: 10.1186/1741-7007-5-57 (PMC2254591; doi:10.1186/1741-7007-5-57)
Supplement: Additional file 11 — Table of summary statistics for microsatellite data (381 specimens, all populations and pelage subspecies) [file 1741-7007-5-57-S11.DOC]

**Additional file 11.** Summary statistics for microsatellite data (381 specimens, all populations and pelage subspecies).

| **Locus** | **Allelic**  **Diversity** | **H_O_** | | **H_E_** | |  |
| --- | --- | --- | --- | --- | --- | --- |
| Neck73 | 12 | 0.487 | | 0.786 | |  |
| **Neck102** | 7 | 0.192 | | 0.419 | |  |
| **Neck447** | 5 | 0.157 | | 0.540 | |  |
| **Neck480** | 11 | 0.282 | | 0.736 | |  |
| **Neck484** | 10 | 0.677 | | 0.652 | |  |
| **Neck550** | 16 | 0.405 | | 0.864 | |  |
| **Neck561** | 12 | 0.444 | | 0.752 | |  |
| **Neck562** | 11 | 0.411 | | 0.840 | |  |
| **Neck567** | 16 | 0.322 | | 0.541 | |  |
| **Neck582** | 4 | 0.661 | | 0.667 | |  |
| **Neck626** | 11 | 0.624 | | 0.790 | |  |
| **Neck665** | 24 | 0.520 | | 0.874 | |  |
| **Neck835** | 13 | 0.313 | | 0.784 | |  |
| **Neck1004** | 13 | 0.534 | | 0.796 | |  |
| **Average** | **11.8** | | **0.431** | | **0.717** | |
