# Supplementary material for: Extensive population genetic structure in the giraffe
Source: BMC Biol. 2007 Dec 21;5:57. doi: 10.1186/1741-7007-5-57 (PMC2254591; doi:10.1186/1741-7007-5-57)
Supplement: Additional file 12 — Tables showing (A) observed and expected heterozygosity, and deviations from Hardy-Weinberg equilibrium in six giraffe subspecific populations, and (B) observed and expected heterozygosity, and deviations from Hardy-Weinberg equilibrium in 16 giraffe populations [file 1741-7007-5-57-S12.DOC]

# Additional file 12A. Observed and expected heterozygosity, and deviations from Hardy-Weinberg equilibrium (significant deviations indicated by +/-, p<0.005, Bonferroni multiple tests correction) in six giraffe subspecific populations. Minus sign (-) indicates heterozygote deficiency, and plus sign (+) indicates heterozygote excess.

|  | ***G.c.***  ***angolensis***  **Angolan** | | | ***G.c.***  ***giraffa***  South Africa | | | ***G.c.***  ***peralta***  **West Africa** | | | ***G.c.***  ***reticulata***  Reticulated | | | ***G.c.***  ***rothschildi***  Rothschilds | | | ***G.c.***  ***tippelskirchi***  ***Masai*** | | | Global Tests/Summary | | |
| --- | --- | --- | --- | --- | --- | --- | --- | --- | --- | --- | --- | --- | --- | --- | --- | --- | --- | --- | --- | --- | --- |
| Locus | **H_O_** | **H_E_** | **HW**  **Equib** | **H_O_** | **H_E_** | **HW**  **Equib** | **H_O_** | **H_E_** | **HW**  **Equib.** | **H_O_** | **H_E_** | **HW**  **Equib.** | **H_O_** | **H_E_** | **HW Equib.** | **H_O_** | **H_E_** | **HW Equib.** | **Range**  **Of H_O_** | Range **of H_E_** | **HW**  **Equib.** |
| Neck73 | 0.459 | 0.655 | - | 0.154 | 0.142 |  | 0.714 | 0.584 |  | 0.605 | 0.759 |  | 0.733 | 0.752 |  | 0.309 | 0.635 | - | 0.154-0.714 | 0.142-0.759 | - |
| Neck102 | 0.108 | 0.102 |  | 0.192 | 0.240 |  | 0.250 | 0.219 |  | 0.156 | 0.147 |  | 0.184 | 0.239 |  | 0.228 | 0.232 |  | 0.108-0.250 | 0.102-0.240 | - |
| Neck447 | 0.000 | 0.000 |  | 0.000 | 0.530 | - | 0.321 | 0.622 |  | 0.299 | 0.405 | - | 0.368 | 0.426 | - | 0.000 | 0.188 | - | 0-0.368 | 0-0.622 | - |
| Neck480 | 0.054 | 0.053 |  | 0.154 | 0.210 |  | 0.240 | 0.480 |  | 0.500 | 0.788 | - | 0.368 | 0.547 |  | 0.206 | 0.398 | - | 0.054-0.500 | 0.053-0.788 | - |
| Neck484 | 0.432 | 0.365 |  | 0.923 | 0.497 | + | 0.393 | 0.357 |  | 0.818 | 0.546 |  | 0.539 | 0.714 | - | 0.752 | 0.531 |  | 0.393-0.923 | 0.357-0.714 | - |
| Neck550 | 0.351 | 0.669 |  | 0.077 | 0.489 |  | 0.536 | 0.799 | - | 0.623 | 0.800 | - | 0.240 | 0.457 |  | 0.423 | 0.744 |  | 0.077-0.623 | 0.457-0.800 | - |
| Neck561 | 0.568 | 0.619 |  | 0.038 | 0.578 | - | 0.481 | 0.529 |  | 0.750 | 0.749 |  | 0.066 | 0.102 | - | 0.523 | 0.709 | - | 0.038-0.750 | 0.102-0.749 | - |
| Neck562 | 0.162 | 0.199 |  | 0.462 | 0.603 |  | 0.286 | 0.595 | - | 0.481 | 0.727 | - | 0.276 | 0.680 | - | 0.529 | 0.697 | - | 0.162-0.529 | 0.199-0.727 | - |
| Neck567 | 0.108 | 0.151 |  | 0.038 | 0.075 |  | 0.074 | 0.137 |  | 0.740 | 0.731 |  | 0.267 | 0.613 |  | 0.276 | 0.312 | - | 0.038-0.740 | 0.075-0.731 | - |
| Neck582 | 0.973 | 0.538 | + | 0.038 | 0.421 | - | 0.679 | 0.614 |  | 0.753 | 0.668 |  | 0.947 | 0.633 | + | 0.482 | 0.617 |  | 0.038-0.973 | 0.421-0.668 |  |
| Neck626 | 0.297 | 0.407 |  | 0.731 | 0.743 |  | 0.750 | 0.511 |  | 0.688 | 0.678 | - | 0.395 | 0.484 | - | 0.757 | 0.795 | - | 0.297-0.757 | 0.407-0.795 | - |
| Neck665 | 0.270 | 0.435 |  | 0.154 | 0.686 | - | 0.607 | 0.786 |  | 0.727 | 0.843 | - | 0.461 | 0.806 | - | 0.555 | 0.860 | - | 0.154-0.727 | 0.435-0.860 | - |
| Neck835 | 0.649 | 0.537 |  | 0.038 | 0.211 | - | 0.714 | 0.739 |  | 0.390 | 0.766 | - | 0.307 | 0.549 |  | 0.153 | 0.664 | - | 0.038-0.714 | 0.211-0.766 | - |
| Neck1004 | 0.108 | 0.103 |  | 0.731 | 0.697 |  | 0.250 | 0.344 |  | 0.727 | 0.811 | - | 0.421 | 0.596 |  | 0.625 | 0.825 | - | 0.108-0.731 | 0.103-0.825 | - |
| Average | 0.324 | 0.345 |  | 0.266 | 0.437 |  | 0.450 | 0.523 |  | 0.590 | 0.673 |  | 0.398 | 0.543 |  | 0.416 | 0.586 |  |  |  |  |

**Additional file 12B.** Observed and expected heterozygosity, and deviations from Hardy-Weinberg equilibrium (significant deviations indicated by +/-, p<0.05, Bonferroni multiple tests correction) in sixteen giraffe populations. Minus sign (-) indicates heterozygote deficiency, and plus sign (+) indicates heterozygote excess.

| **South African giraffe (*G.c. giraffa*)** | | | |
| --- | --- | --- | --- |
| Locus | **H_O_** | **H_E_** | **HW**  **Equib.** |
| Neck73 | 0.154 | 0.142 |  |
| Neck102 | 0.192 | 0.240 |  |
| Neck447 | 0.000 | 0.530 | - |
| Neck480 | 0.154 | 0.210 |  |
| Neck484 | 0.923 | 0.497 | + |
| Neck550 | 0.077 | 0.489 |  |
| Neck561 | 0.038 | 0.578 | - |
| Neck562 | 0.462 | 0.603 |  |
| Neck567 | 0.038 | 0.075 |  |
| Neck582 | 0.038 | 0.421 | - |
| Neck626 | 0.731 | 0.743 |  |
| Neck665 | 0.154 | 0.686 | - |
| Neck835 | 0.038 | 0.211 | - |
| Neck1004 | 0.731 | 0.697 |  |
| Average | 0.266 | 0.437 |  |

| **West African giraffe (*G.c. peralta*)** | | | |
| --- | --- | --- | --- |
| Locus | **H_O_** | **H_E_** | **HW**  **Equib.** |
| Neck73 | 0.714 | 0.584 |  |
| Neck102 | 0.250 | 0.219 |  |
| Neck447 | 0.321 | 0.622 |  |
| Neck480 | 0.240 | 0.480 |  |
| Neck484 | 0.393 | 0.357 |  |
| Neck550 | 0.536 | 0.799 |  |
| Neck561 | 0.481 | 0.529 |  |
| Neck562 | 0.286 | 0.595 | - |
| Neck567 | 0.074 | 0.137 |  |
| Neck582 | 0.679 | 0.614 |  |
| Neck626 | 0.750 | 0.511 |  |
| Neck665 | 0.607 | 0.786 |  |
| Neck835 | 0.714 | 0.739 |  |
| Neck1004 | 0.250 | 0.344 |  |
| Average | 0.450 | 0.523 |  |

|  | **Rothschild’s giraffe (*G.c. rothschildi*)**  Uganda Kenya | | | | | | Populations  Summary | | |
| --- | --- | --- | --- | --- | --- | --- | --- | --- | --- |
| Locus | **H_O_** | **H_E_** | **HW**  **Equib** | **H_O_** | **H_E_** | **HW Equib** | **Range**  **of H_O_** | Range **of H_E_** |  |
| Neck73 | 0.760 | 0.597 |  | 0.680 | 0.690 |  | 0.680-0.760 | 0.597-0.690 |  |
| Neck102 | 0.275 | 0.339 |  | 0.000 | 0.000 |  | 0-0.275 | 0-0.339 |  |
| Neck447 | 0.412 | 0.450 |  | 0.280 | 0.358 |  | 0.280-0.412 | 0.358-0.450 |  |
| Neck480 | 0.333 | 0.511 |  | 0.440 | 0.602 |  | 0.333-0.440 | 0.511-0.602 |  |
| Neck484 | 0.314 | 0.662 | - | 1.000 | 0.567 |  | 0.314-1.000 | 0.567-0.662 |  |
| Neck550 | 0.216 | 0.433 | - | 0.292 | 0.498 |  | 0.216-0.292 | 0.433-0.498 |  |
| Neck561 | 0.098 | 0.113 |  | 0.000 | 0.077 |  | 0.000-0.098 | 0.077-0.113 |  |
| Neck562 | 0.333 | 0.539 |  | 0.160 | 0.343 |  | 0.160-0.333 | 0.343-0.539 |  |
| Neck567 | 0.360 | 0.385 |  | 0.080 | 0.218 |  | 0-60 | 0.218-0.385 |  |
| Neck582 | 0.922 | 0.641 | + | 1.000 | 0.611 |  | 0.922-1.000 | 0.611-0.641 |  |
| Neck626 | 0.431 | 0.504 |  | 0.320 | 0.438 |  | 0.320-0.431 | 0.438-0.504 |  |
| Neck665 | 0.510 | 0.756 | - | 0.360 | 0.462 |  | 0.360-0.510 | 0.462-0.756 |  |
| Neck835 | 0.294 | 0.407 |  | 0.333 | 0.360 |  | 0.294-0.333 | 0.360-0.407 |  |
| Neck1004 | 0.373 | 0.370 |  | 0.520 | 0.551 |  | 0.373-0.520 | 0.370-0.551 |  |
| Average | 0.402 | 0.479 |  | 0.390 | 0.412 |  |  |  |  |

|  | | **Reticulated giraffe (*G.c. reticulata*)**  Meru N.P. Samburu N.R. Laikipia | | | | | | | | | | Populations  Summary | |
| --- | --- | --- | --- | --- | --- | --- | --- | --- | --- | --- | --- | --- | --- |
| Locus | **H_O_** | | **H_E_** | **HW**  **Equib** | **H_O_** | **H_E_** | **HW Equib** | **H_O_** | **H_E_** | **HW Equib** | **Range**  **of H_O_** | | Range **of H_E_** |
| Neck73 | 0.800 | | 0.760 |  | 0.500 | 0.801 |  | 0.600 | 0.635 |  | 0.500-0.800 | | 0.500-0.760 |
| Neck102 | 0.000 | | 0.000 |  | 0.294 | 0.258 |  | 0.140 | 0.130 |  | 0-0.294 | | 0-0.258 |
| Neck447 | 0.100 | | 0.095 |  | 0.118 | 0.304 |  | 0.400 | 0.473 |  | 0.100-0.400 | | 0.095-0.473 |
| Neck480 | 0.500 | | 0.410 |  | 0.500 | 0.738 |  | 0.500 | 0.650 |  | 0.500 | | 0.410-0.738 |
| Neck484 | 0.900 | | 0.535 |  | 0.882 | 0.538 |  | 0.780 | 0.550 |  | 0.780-0.900 | | 0.535-0.550 |
| Neck550 | 0.300 | | 0.420 |  | 0.471 | 0.599 |  | 0.740 | 0.786 |  | 0.300-0.740 | | 0.420-0.786 |
| Neck561 | 0.556 | | 0.660 |  | 0.706 | 0.779 |  | 0.800 | 0.689 |  | 0.556-0.800 | | 0.660-0.779 |
| Neck562 | 0.200 | | 0.640 |  | 0.353 | 0.645 |  | 0.580 | 0.719 |  | 0.200-0.580 | | 0.640-0.719 |
| Neck567 | 0.400 | | 0.650 |  | 0.706 | 0.649 |  | 0.820 | 0.734 |  | 0.400-0.820 | | 0.649-0.734 |
| Neck582 | 0.800 | | 0.640 |  | 0.824 | 0.630 |  | 0.720 | 0.671 |  | 0.720-0.800 | | 0.630-0.671 |
| Neck626 | 0.700 | | 0.720 |  | 0.588 | 0.718 |  | 0.720 | 0.640 |  | 0.588-0.720 | | 0.640-0.720 |
| Neck665 | 0.400 | | 0.465 |  | 0.765 | 0.817 |  | 0.780 | 0.807 |  | 0.400-0.780 | | 0.465-0.817 |
| Neck835 | 0.500 | | 0.740 |  | 0.235 | 0.754 | **-** | 0.420 | 0.705 | **-** | 0.235-0.500 | | 0.705-0.740 |
| Neck1004 | 0.700 | | 0.770 |  | 0.706 | 0.825 |  | 0.740 | 0.752 |  | 0.700-0.740 | | 0.752-0.825 |
| Average | 0.490 | | 0.536 |  | 0.546 | 0.647 |  | 0.624 | 0.639 |  |  | |  |

|  | | **Angolan giraffe (*G.c. angolensis*)**  Etosha Hoanib River Khumib River | | | | | | | | | | Populations  Summary | |
| --- | --- | --- | --- | --- | --- | --- | --- | --- | --- | --- | --- | --- | --- |
| Locus | **H_O_** | | **H_E_** | **HW**  **Equib** | **H_O_** | **H_E_** | **HW Equib** | **H_O_** | **H_E_** | **HW Equib** | **Range**  **of H_O_** | | Range **of H_E_** |
| Neck73 | 0.600 | | 0.571 |  | 0.400 | 0.678 |  | 0.250 | 0.531 |  | 0.250-0.600 | | 0.531-0.571 |
| Neck102 | 0.133 | | 0.124 |  | 0.133 | 0.124 |  | 0.000 | 0.000 |  | 0-0.133 | | 0-0.124 |
| Neck447 | 0.000 | | 0.000 |  | 0.000 | 0.000 |  | 0.000 | 0.000 |  | 0-0 | | 0-0 |
| Neck480 | 0.000 | | 0.000 |  | 0.133 | 0.124 |  | 0.000 | 0.000 |  | 0-0.133 | | 0-0.124 |
| Neck484 | 0.400 | | 0.382 |  | 0.600 | 0.420 |  | 0.000 | 0.000 |  | 0-0.600 | | 0-0.420 |
| Neck550 | 0.600 | | 0.724 |  | 0.267 | 0.624 |  | 0.000 | 0.375 |  | 0-0.600 | | 0.375-0.724 |
| Neck561 | 0.667 | | 0.669 |  | 0.667 | 0.589 |  | 0.250 | 0.406 |  | 0.250-0.667 | | 0.406-0.669 |
| Neck562 | 0.133 | | 0.238 |  | 0.200 | 0.184 |  | 0.250 | 0.219 |  | 0.133-0.250 | | 0.184-0.219 |
| Neck567 | 0.067 | | 0.064 |  | 0.067 | 0.180 |  | 0.000 | 0.000 |  | 0-0.067 | | 0-0.180 |
| Neck582 | 0.933 | | 0.531 |  | 1.000 | 0.558 |  | 1.000 | 0.500 |  | 0.933-1.000 | | 0.500-0.558 |
| Neck626 | 0.400 | | 0.560 |  | 0.067 | 0.127 |  | 0.500 | 0.406 |  | 0.067-0.500 | | 0.127-0.560 |
| Neck665 | 0.333 | | 0.527 |  | 0.200 | 0.291 |  | 0.500 | 0.406 |  | 0.200-0.500 | | 0.291-0.527 |
| Neck835 | 0.600 | | 0.438 |  | 0.667 | 0.589 |  | 0.500 | 0.375 |  | 0.500-0.667 | | 0.375-0.589 |
| Neck1004 | 0.067 | | 0.064 |  | 0.133 | 0.124 |  | 0.250 | 0.219 |  | 0.067-0.250 | | 0.064-0.219 |
| Average | 0.352 | | 0.350 |  | 0.324 | 0.330 |  | 0.250 | 0.246 |  |  | |  |

|  | **Masai giraffe (*G.c. tippelskirchi*)**  **Athi River Chyulu Hills Serengeti NP Tarangire NP Manyara NP Lake Naivasha** | | | | | | | | | | | | | | | | | | Populations  Summary | | |
| --- | --- | --- | --- | --- | --- | --- | --- | --- | --- | --- | --- | --- | --- | --- | --- | --- | --- | --- | --- | --- | --- |
| Locus | **H_O_** | **H_E_** | **HW**  **Equib.** | **H_O_** | **H_E_** | **HW**  **Equib.** | **H_O_** | **H_E_** | **HW**  **Equib.** | **H_O_** | **H_E_** | **HW**  **Equib.** | **H_O_** | **H_E_** | **HW Equib.** | **H_O_** | **H_E_** | **HW Equib.** | **Range**  **Of H_O_** | Range **of H_E_** |  |
| Neck73 | 0.440 | 0.502 |  | 0.240 | 0.458 |  | 0.360 | 0.647 |  | 0.118 | 0.471 |  | 0.333 | 0.517 |  | 0.143 | 0.520 |  | 0.118-0.440 | 0.458-0.647 |  |
| Neck102 | 0.160 | 0.150 |  | 0.200 | 0.241 |  | 0.120 | 0.150 |  | 0.588 | 0.415 |  | 0.500 | 0.375 |  | 0.000 | 0.000 |  | 0-0.588 | 0-0.415 |  |
| Neck447 | 0.000 | 0.269 | **-** | 0.000 | 0.509 | **-** | 0.000 | 0.000 |  | 0.000 | 0.000 |  | 0.000 | 0.000 |  | 0.000 | 0.000 |  | 0-0 | 0-0.509 |  |
| Neck480 | 0.240 | 0.618 |  | 0.320 | 0.452 |  | 0.157 | 0.183 |  | 0.176 | 0.164 |  | 0.083 | 0.080 |  | 0.333 | 0.569 |  | 0.083-0.333 | 0.080-0.618 |  |
| Neck484 | 0.680 | 0.634 |  | 0.840 | 0.510 |  | 0.725 | 0.487 |  | 0.941 | 0.498 |  | 0.667 | 0.444 |  | 0.571 | 0.408 |  | 0.571-0.941 | 0.408-0.634 |  |
| Neck550 | 0.480 | 0.724 |  | 0.080 | 0.078 |  | 0.549 | 0.612 |  | 0.412 | 0.631 |  | 0.417 | 0.656 |  | 0.571 | 0.439 |  | 0.080-0.571 | 0.078-0.724 |  |
| Neck561 | 0.320 | 0.403 |  | 0.520 | 0.608 |  | 0.644 | 0.766 |  | 0.471 | 0.740 |  | 0.500 | 0.573 |  | 0.667 | 0.569 |  | 0.320-0.667 | 0.403-0.766 |  |
| Neck562 | 0.400 | 0.634 |  | 0.880 | 0.650 |  | 0.460 | 0.678 |  | 0.529 | 0.682 |  | 0.583 | 0.705 |  | 0.143 | 0.357 |  | 0.143-0.880 | 0.357-0.705 |  |
| Neck567 | 0.120 | 0.152 |  | 0.200 | 0.327 |  | 0.286 | 0.258 |  | 0.235 | 0.313 |  | 0.636 | 0.603 |  | 0.571 | 0.439 |  | 0.120-0.636 | 0.152-0.603 |  |
| Neck582 | 0.280 | 0.418 |  | 0.120 | 0.218 |  | 0.549 | 0.633 |  | 0.706 | 0.581 |  | 0.833 | 0.656 |  | 0.857 | 0.612 |  | 0.120-0.857 | 0.218-0.656 |  |
| Neck626 | 0.880 | 0.808 |  | 0.800 | 0.822 |  | 0.725 | 0.725 |  | 0.765 | 0.718 |  | 0.818 | 0.661 |  | 0.286 | 0.459 |  | 0.286-0.880 | 0.459-0.822 |  |
| Neck665 | 0.480 | 0.835 | **-** | 0.560 | 0.850 |  | 0.529 | 0.780 | **-** | 0.706 | 0.851 |  | 0.667 | 0.840 |  | 0.429 | 0.745 |  | 0.429-0.706 | 0.745-0.851 |  |
| Neck835 | 0.200 | 0.220 |  | 0.000 | 0.000 |  | 0.118 | 0.704 | **-** | 0.294 | 0.497 |  | 0.250 | 0.594 |  | 0.286 | 0.531 |  | 0-0.294 | 0-0.704 |  |
| Neck1004 | 0.480 | 0.541 |  | 0.560 | 0.724 |  | 0.706 | 0.686 |  | 0.647 | 0.683 |  | 0.636 | 0.661 |  | 0.714 | 0.622 |  | 0.480-0.714 | 0.541-0.724 |  |
| Average | 0.369 | 0.493 |  | 0.380 | 0.460 |  | 0.423 | 0.522 |  | 0.471 | 0.518 |  | 0.495 | 0.526 |  | 0.398 | 0.448 |  |  |  |  |
