# Supplementary material for: Extensive population genetic structure in the giraffe
Source: BMC Biol. 2007 Dec 21;5:57. doi: 10.1186/1741-7007-5-57 (PMC2254591; doi:10.1186/1741-7007-5-57)
Supplement: Additional file 13 — Tables showing (A) posterior probability population assignments of 381 Individuals, based on assignment to pelage/subspecies designations using STRUCTURE [19], and (B) subspecies assignment of 381 individuals, based on assignment to pelage/subspecies designations, using multilocus genotypes and Bayesian analysis (Rannala and Mountain method in Geneclass2 [46,47]) [file 1741-7007-5-57-S13.DOC]

**Additional file 13A.** Posterior probability population assignments of 381 Individuals, based on assignment to pelage/subspecies designations using STRUCTURE [19].

| **Assignment**  **Probability** | **Quantity** | **% of**  **Individuals** |  |
| --- | --- | --- | --- |
| >0.99 | 319 | 83.7 |  |
| >0.95 | 365 | 95.8 |  |
| >0.90 | 371 | 97.4 |  |
| >0.80 | 375 | 98.4 |  |
| >0.70 | 378 | 99.2 |  |
| 0.624 | 1 | (Ind. GR41) |  |
| 0.248 | 1 | (Ind. GS43) |  |
| 0.179 | 1 | (Ind. 4826) |  |

**Additional file 13B.** Subspecies assignment of 381 Individuals, based on assignment to pelage/subspecies designations, using multilocus genotypes and Bayesian analysis (Rannala and Mountain method in Geneclass2 [46, 47]).

| **Assignment**  **Probability** | **Quantity** | **% of**  **Individuals** |  |
| --- | --- | --- | --- |
| >0.995 | 377 | 99.0 |  |
| >0.990 | 379 | 99.5 |  |
| 0.976 | 1 | (Ind GS43) |  |
| 0.625 | 1 | (Ind. G8) |  |
