# Supplementary material for: Extensive population genetic structure in the giraffe
Source: BMC Biol. 2007 Dec 21;5:57. doi: 10.1186/1741-7007-5-57 (PMC2254591; doi:10.1186/1741-7007-5-57)
Supplement: Additional file 14 — Table of STRUCTURE [23] cluster results identify three possible subspecies hybrids, four population hybrids within the same subspecies and one possible population migrant within the same subspecies [file 1741-7007-5-57-S14.DOC]

**Additional file 14.** STRUCTURE [23] cluster results identify three possible subspecies hybrids, four population hybrids within the same subspecies and one possible population migrant within the same subspecies.

###### Subspecies Hybrids

| **Possible Subspecies Hybrid** | **Subspecies Sampling Origin** | **Cluster**  **Assignment** |
| --- | --- | --- |
| GR41 | Rothschilds | Rothschilds 74% / Reticulated 16% |
| GS43 | Reticulated | Reticulated 51% / Rothschilds 42% |
| 4826 | Masai | Masai 63% / Reticulated 23% |

Population Hybrids

| **Possible Population Hybrid** | **Population Sampling Origin** | **Cluster**  **Assignment** |
| --- | --- | --- |
| 4811 | Reticulated – Samburu Nat. Res. | Samburu 66% / Meru Nat. Park 14% |
| 4880 | Masai – Tanzania | Tanzania 63% / Kenya 34% |
| GirP1 | Reticulated – Laikipia Region | Laikipia 65% / Meru Nat. Park 30% |
| GirR21 | Reticulated – Laikipia Region | Laikipia 55% / Meru Nat. Park 38% |

Population Migrant

| **Possible Population Migrant** | **Population Sampling Origin** | | **Cluster**  **Assignment** |
| --- | --- | --- | --- |
| GS44 | Reticulated – Laikipia Region | Samburu National Reserve | |
