# Supplementary material for: Extensive population genetic structure in the giraffe
Source: BMC Biol. 2007 Dec 21;5:57. doi: 10.1186/1741-7007-5-57 (PMC2254591; doi:10.1186/1741-7007-5-57)

**Additional file 15.** STRUCTURE [19] cluster assignments detect: a) three possible subspecies hybrids, b) four population hybrids within the same subspecies, and c) a population migrant within the same species for the three subspecies whose geographic ranges adjoin each other in east Africa (*G.c. rothschildi*, *G.c. reticulata*, and *G.c. tippleskirchi*).


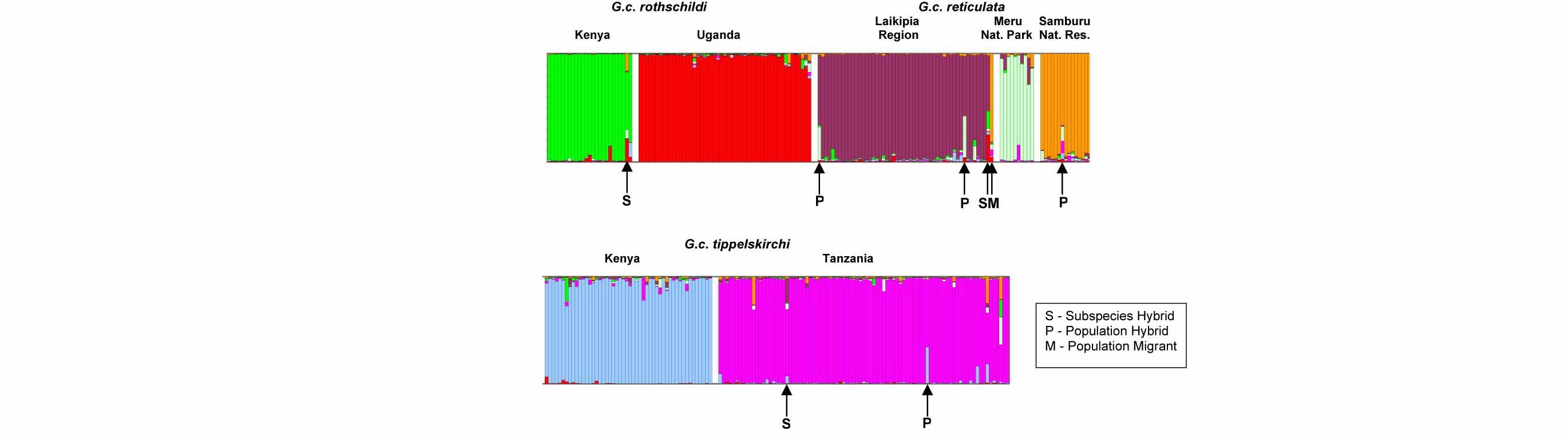

Supplement: Additional file 15 — Figure showing STRUCTURE [19] cluster assignments of detected giraffe hybrids [file 1741-7007-5-57-S15.DOC]
