# Supplementary material for: Extensive population genetic structure in the giraffe
Source: BMC Biol. 2007 Dec 21;5:57. doi: 10.1186/1741-7007-5-57 (PMC2254591; doi:10.1186/1741-7007-5-57)
Supplement: Additional file 16 — Tables showing (A) pairwise Fst values and statistical significance for the six giraffe subspecies; (B) pairwise values for Nei's genetic distance among six giraffe subspecies; (C) G.c. angolensis (Angolan) population pairwise comparison Fst values and statistical significance; (D) G.c. reticulata (Reticulated) population pairwise comparison Fst values and statistical significance; (E) G.c. rothschildi (Rothschild's) population pairwise comparison Fst values and statistical significance; (F) G.c. tippelskirchi (Masai) population pairwise comparison Fst values and statistical significance; and (G) G.c. tippelskirchi (Masai) Serengeti N.P. population pairwise comparison Fst values and statistical significance [file 1741-7007-5-57-S16.DOC]

#### Additional file 16A. Pairwise F_st_ values (below diagonal) and statistical significance (above diagonal; asterisk indicates *P* < 0.05, G test) for the six giraffe subspecies.

| ***G.c.***  ***angolensis*** | ***G.c.***  ***giraffa*** | ***G.c.***  ***peralta*** | ***G.c. reticulata*** | ***G.c. rothschildi*** | ***G.c. tippelskirchi*** |  |
| --- | --- | --- | --- | --- | --- | --- |
| --- | * | * | * | * | * | **G.c. angolensis** |
| 0.352 | --- | * | * | * | * | ***G.c. giraffa*** |
| 0.452 | 0.403 | --- | * | * | * | ***G.c. peralta*** |
| 0.338 | 0.294 | 0.198 | --- | * | * | ***G.c. reticulata*** |
| 0.466 | 0.432 | 0.236 | 0.203 | --- | * | ***G.c. rothschildi*** |
| 0.307 | 0.251 | 0.231 | 0.113 | 0.269 | --- | ***G.c. tippelskirchi*** |

**Additional file** **16B.** Pairwise values for Nei’s genetic distance among six giraffe subspecies.

| ***G.c.***  ***angolensis*** | ***G.c.***  ***giraffa*** | ***G.c.***  ***peralta*** | ***G.c. reticulata*** | ***G.c. rothschildi*** | ***G.c. tippelskirchi*** |  |
| --- | --- | --- | --- | --- | --- | --- |
| --- |  |  |  |  |  | **G.c. angolensis** |
| 0.445 | --- |  |  |  |  | ***G.c. giraffa*** |
| 1.002 | 1.077 | --- |  |  |  | ***G.c. peralta*** |
| 0.874 | 0.887 | 0.518 | --- |  |  | ***G.c. reticulata*** |
| 1.453 | 1.578 | 0.473 | 0.515 | --- |  | ***G.c. rothschildi*** |
| 0.585 | 0.510 | 0.524 | 0.246 | 0.676 | --- | ***G.c. tippelskirchi*** |

**Additional file** **16C.** *G.c. angolensis* (Angolan) population pairwise comparison Fst values (below diagonal) and statistical significance (above diagonal; asterisk indicates *P* < 0.05, G test).

| **Etosha** | **Hoanib River** | **Khumib River** |  |
| --- | --- | --- | --- |
| --- | * | NS | **Etosha** |
| 0.044 | --- | NS | **Hoanib River** |
| 0.012 | 0.031 | --- | **Khumib River** |

**Additonal file 16D.** *G.c. reticulata* (Reticulated) population pairwise comparison Fst values (below diagonal) and statistical significance (above diagonal; asterisk indicates *P* < 0.05, G test).

| **Meru N.P.** | **Laikipia** | **Samburu N.R.** |  |
| --- | --- | --- | --- |
| --- | * | * | **Meru N.P.** |
| 0.113 | --- | * | **Laikipia** |
| 0.104 | 0.093 | --- | **Samburu N.R.** |

**Additional file 16E.** *G.c. rothschildi* (Rothschild’s) population pairwise comparison Fst values (below diagonal) and statistical significance (above diagonal; asterisk indicates *P* < 0.05, G test).

| **Kenya** | **Uganda** |  |
| --- | --- | --- |
| --- | * | **Kenya** |
| 0.286 | --- | **Uganda** |

**Additional file 16F.** *G.c. tippelskirchi* (Masai) population pairwise comparison Fst values (below diagonal) and statistical significance (above diagonal; asterisk indicates *P* < 0.05, G test).

| Athi | **Chyulu** | **Serengeti N.P.** | **Tarangire N.P.** | **Manyara N.P.** | **Lake Naivasha** |  |
| --- | --- | --- | --- | --- | --- | --- |
| --- | * | * | * | * | * | **Athi** |
| 0.077 | --- | * | * | * | * | **Chyulu** |
| 0.184 | 0.220 | --- | * | * | * | **Serengeti N.P.** |
| 0.207 | 0.235 | 0.059 | --- | NS | * | **Tarangire N.P.** |
| 0.188 | 0.228 | 0.038 | 0.030 | --- | * | **Manyara N.P.** |
| 0.190 | 0.242 | 0.116 | 0.136 | 0.094 | --- | **Lake Naivasha** |

**Additional file 16G.** *G.c. tippelskirchi* (Masai) Serengeti N.P. population pairwise comparison Fst values (below diagonal) and statistical significance (above diagonal; asterisk indicates *P* < 0.05, G test).

| Grumeti | **Kirawira** | **Ndutu** | **Seronera** | **Varicho** |  |
| --- | --- | --- | --- | --- | --- |
| --- | NS | NS | NS | NS | Grumeti |
| 0.038 | --- | * | * | NS | Kirawira |
| 0.103 | 0.121 | --- | NS | NS | **Ndutu** |
| 0.097 | 0.126 | -0.001 | --- | * | **Seronera** |
| 0.033 | 0.023 | 0.059 | 0.080 | --- | **Varicho** |
