# Supplementary material for: Extensive population genetic structure in the giraffe
Source: BMC Biol. 2007 Dec 21;5:57. doi: 10.1186/1741-7007-5-57 (PMC2254591; doi:10.1186/1741-7007-5-57)
Supplement: Additional file 17 — Table showing migration rates among giraffe subspecies [file 1741-7007-5-57-S17.DOC]

**Additional file 17.** Migration rates among giraffe subspecies. Means of the posterior distributions of m, the migration rate into each position, are shown. The populations from which each individual was sampled are listed in the rows, while the populations from which they migrated are listed in the columns. Values along the diagonal are the proportions of individuals derived from the source populations each generation. Standard deviation for all distributions were < 0.05.

|  | ***G.c. peralta*** | ***G.c. rothschildi*** | ***G.c. reticulata*** | ***G.c. tippleskirchi*** | ***G.c. giraffa*** | ***G.c. angolensis*** |
| --- | --- | --- | --- | --- | --- | --- |
| *G.c. peralta* | **0.99** | 0.00 | 0.00 | 0.00 | 0.00 | 0.00 |
| ***G.c. rothschildi*** | 0.00 | **1.00** | 0.00 | 0.00 | 0.00 | 0.00 |
| ***G.c. reticulata*** | 0.00 | 0.00 | **1.00** | 0.00 | 0.00 | 0.00 |
| ***G.c. tippleskirchi*** | 0.00 | 0.00 | 0.00 | **1.00** | 0.00 | 0.00 |
| ***G.c. giraffa*** | 0.00 | 0.00 | 0.00 | 0.00 | **0.99** | 0.00 |
| ***G.c. angolensis*** | 0.00 | 0.00 | 0.00 | 0.00 | 0.00 | **0.99** |
