# Supplementary material for: Extensive population genetic structure in the giraffe
Source: BMC Biol. 2007 Dec 21;5:57. doi: 10.1186/1741-7007-5-57 (PMC2254591; doi:10.1186/1741-7007-5-57)
Supplement: Additional file 18 — Table of primer sequences and amplification characteristics of Giraffa camelopardalis microsatellite locus NECK484 [file 1741-7007-5-57-S18.DOC]

**Additional file 18.** Primer sequences and amplification characteristics of *Giraffa camelopardalis* microsatellite locus NECK484.

| **Locus** | **Primer Sequence** | **Repeat Motif** | **Dye Label** | **Annealing Temp. (^o^C)** | **Genbank Accession No.** |
| --- | --- | --- | --- | --- | --- |
| 11HDZ484 | F: GCC TGG GGG AGC TAG AGT C | (CA)_2_(CG)_2_(CA)_6_ | HEX | 52 | AY727871 |
|  | R: AAC TCA GAT TGC CTT GCC C |  |  |  |  |
