# Supplementary material for: Extensive population genetic structure in the giraffe
Source: BMC Biol. 2007 Dec 21;5:57. doi: 10.1186/1741-7007-5-57 (PMC2254591; doi:10.1186/1741-7007-5-57)
Supplement: Additional file 19 — Figure showing likelihood values for inferred number of genetic clusters (K) from STRUCTURE [23] (three iterations per value of K) [file 1741-7007-5-57-S19.DOC]

**Additional file 19.** Likelihood values for inferred number of genetic clusters (K) from STRUCTURE [23] 3 iterations per value of K).

| **K** | **Avg. Ln P(D)** |
| --- | --- |
| 1 | -17453 |
| 2 | -15390 |
| 3 | -14271 |
| 4 | -13165 |
| 5 | -12610 |
| 6 | -12126 |
| 7 | -11915 |
| 8 | -11932 |
| 9 | -11444 |
| 10 | -11392 |
| 11 | -11337 |
| 12 | -11239 |
| 13 | -10952 |
| 14 | -11300 |
| 15 | -11121 |
| 16 | -10838 |
