# Supplementary material for: Extensive population genetic structure in the giraffe
Source: BMC Biol. 2007 Dec 21;5:57. doi: 10.1186/1741-7007-5-57 (PMC2254591; doi:10.1186/1741-7007-5-57)
Supplement: Additional file 20 — Table of overall Fis values per subspecies, and per population [file 1741-7007-5-57-S20.DOC]

**Additional file 20.** Overall F_is_ values per subspecies, and per population. Asterisks indicate significance at the 0.05 level.

| **Subspecies** | **Fis** **(Genepop)** | **Fis** **(BayesAss)** | **Significant (α=0.05)** |
| --- | --- | --- | --- |
| G.c. angolensis | 0.074 | 0.172 | * |
| *G.c. giraffa* | 0.407 | 0.483 | * |
| *G.c. peralta* | 0.157 | 0.222 | * |
| *G.c. reticulata* | 0.130 | 0.172 | * |
| *G.c. rothschildi* | 0.273 | 0.291 | * |
| *G.c. tippelskirchi* | 0.294 | 0.308 | * |

| **Subspecies** | **Population** | **Fis** |
| --- | --- | --- |
| G.c. angolensis | Etosha NP | 0.186 |
|  | Hoanib River | 0.138 |
|  | Khumib River | 0.180 |
|  |  |  |
| *G.c. giraffa* |  | 0.392 |
|  |  |  |
| *G.c. peralta* |  | 0.155 |
|  |  |  |
| *G.c. reticulata* | Laikipia | 0.090 |
|  | Meru NP | 0.164 |
|  | Samburu NP | 0.225 |
|  |  |  |
| *G.c. rothschildi* | Kenya | 0.170 |
|  | Uganda | 0.202 |
|  |  |  |
| *G.c. tippelskirchi* | Athi River | 0.319 |
|  | Chyulu Hills | 0.255 |
|  | Serengeti NP | 0.259 |
|  | Manyara NP | 0.193 |
|  | Lake Naivasha | 0.180 |
|  | Tarangire NP | 0.230 |
