# Supplementary material for: Extensive population genetic structure in the giraffe
Source: BMC Biol. 2007 Dec 21;5:57. doi: 10.1186/1741-7007-5-57 (PMC2254591; doi:10.1186/1741-7007-5-57)
Supplement: Additional file 21 — Table of Mantel test of isolation by distance results (correlation of genetic distance (Fst/(1-Fst) with geographic distance) [file 1741-7007-5-57-S21.DOC]

**Additional file 21.** Mantel test of isolation by distance (correlation of genetic distance (Fst/(1-Fst) with geographic distance). Double asterisks indicate significance at *P* < 0.01.

R^2^  Probability

All Six Subspecies 0.275 0.099

Angolan (*G.c. angolensis*) populations 0.080 0.513

#### Reticulated (*G.c. reticulate*) populations 0.764 0.166

All Masai (*G.c. tippelskirchi*) populations 0.254 0.028 **

Masai (*G.c. tippelskirchi*) Serengeti locations 0.365 0.011 **
