# Supplementary material for: Extensive population genetic structure in the giraffe
Source: BMC Biol. 2007 Dec 21;5:57. doi: 10.1186/1741-7007-5-57 (PMC2254591; doi:10.1186/1741-7007-5-57)
Supplement: Additional file 22 — Table of AMOVA using microsatellite data results [file 1741-7007-5-57-S22.DOC]

**Additional file 22.** Analysis of molecular variance (AMOVA) using microsatellite data.

ANALYSIS – GROUPS (SUBSETS OF THE 6 SUBSPECIES)

AND POPULATIONS (10)

**Group Components (6) Populations (1O)**

# Namibian (G.c. angolensis) Namibia

South African *(G.c. giraffa)* South Africa

West African *(G.c. peralta)* Niger

Reticulated *(G.c. reticulata)* Laikipia*,* Meru N.P., Samburu N.P.

Rothschilds *(G.c. rothschildi)* Kenya, Uganda

Masai *(G.c. tippelskirchi)* Kenya, Tanzania

| Grouping tested | df | SS | Variance component | % of variance |
| --- | --- | --- | --- | --- |
| [per][rot][ret][tip][gir][ang] |  |  |  |  |
| Among Groups [Ф_ct_] | 5 | 1698.54 | 3.44 | 22% |
| Among Populations [Ф_sc_] | 4 | 487.98 | 3.32 | 22% |
| Within Populations [Ф_st_] | 371 | 3212.20 | 8.66 | 56% |
| [per][rot+ret+tip][gir+ang] |  |  |  |  |
| Among Groups [Ф_ct_] | 2 | 735.72 | 2.503 | 15% |
| Among Populations [Ф_sc_] | 7 | 1450.79 | 5.251 | 32% |
| Within Populations [Ф_st_] | 371 | 3212.19 | 8.658 | 53% |
| [per][rot+ret+tip][gir][ang] |  |  |  |  |
| Among Groups [Ф_ct_] | 3 | 923.77 | 2.596 | 16% |
| Among Populations [Ф_sc_] | 6 | 1262.75 | 5.170 | 31% |
| Within Populations [Ф_st_] | 371 | 3212.20 | 8.658 | 53% |
| [per+rot+ret+tip+gir][ang] |  |  |  |  |
| Among Groups [Ф_ct_] | 1 | 446.19 | 3.27 | 19% |
| Among Populations [Ф_sc_] | 8 | 1740.33 | 5.71 | 32% |
| Within Populations [Ф_st_] | 371 | 3212.20 | 8.66 | 49% |
| [per+rot+ret][tip+gir][ang] |  |  |  |  |
| Among Groups [Ф_ct_] | 2 | 946.46 | 2.17 | 14% |
| Among Populations [Ф_sc_] | 7 | 1240.06 | 4.92 | 31% |
| Within Populations [Ф_st_] | 371 | 3212.20 | 8.66 | 55% |
| [per+rot+ret+tip][gir+ang] |  |  |  |  |
| Among Groups [Ф_ct_] | 1 | 558.21 | 3.42 | 20% |
| Among Populations [Ф_sc_] | 8 | 1628.30 | 5.28 | 30% |
| Within Populations [Ф_st_] | 371 | 3212.20 | 8.66 | 50% |
